# Time Series Genomics of Pseudomonas aeruginosa Reveals the Emergence of a Hypermutator Phenotype and Within-Host Evolution in Clinical Inpatients

**DOI:** 10.1128/spectrum.00057-22

**Published:** 2022-07-21

**Authors:** Hongjie Liu, Lang Yang, Qichao Chen, Hongbin Song, Xiaochen Bo, Jingyu Guo, Peng Li, Ming Ni

**Affiliations:** a Institute of Health Service and Transfusion Medicine, Beijing, China; b Chinese PLA Center for Disease Control and Prevention, Beijing, China; c The 316th Hospital of Chinese PLA, Beijing, China; Emory University School of Medicine

**Keywords:** *Pseudomonas aeruginosa*, nosocomial infections, longitudinal study, whole-genome sequencing, hypermutator

## Abstract

Pseudomonas aeruginosa, a common opportunistic pathogen, is one of the leading etiological agents of nosocomial infections. Many previous studies have reported the nosocomial transmission and epidemiology of P. aeruginosa infections. However, longitudinal studies regarding the dynamics of P. aeruginosa colonization and infection in health care settings are limited. We obtained longitudinal samples from aged patients with prolonged intensive care unit (ICU) stays (~4 to 19 months). P. aeruginosa was isolated from 71 samples obtained from seven patients and characterized by whole-genome sequencing. The P. aeruginosa isolates were assigned to 10 clonal complexes, and turnover of main clones was observed in sequential sputum samples from two patients. By comparing intraclonal genomic diversities, we identified two clones that had significantly higher numbers of single nucleotide polymorphisms and variations in homopolymeric sequences than the other clones, indicating a hypermutator phenotype. These hypermutator clones were associated with mutations T147I/G521S and P27L in the MutL protein, and their mutation rates were estimated to be 3.20 × 10^−5^ and 6.59 × 10^−5^ per year per nucleotide, respectively. We also identified 24 recurrently mutated genes that exhibited intraclonal diversity in two or more clones. Notably, one recurrent mutation, S698F in FptA, was observed in four clones. These findings suggest that convergent microevolution and adaption of P. aeruginosa occur in long-term ICU patients.

**IMPORTANCE** Pseudomonas aeruginosa is a predominant opportunistic pathogen that causes nosocomial infections. Inappropriate empirical therapy can lead to prolonged hospital stays and increased mortality. In our study of sequential P. aeruginosa isolates from inpatients, high intrahost diversity was observed, including switching of clones and the emergence of a hypermutator phenotype. Recurrently mutated genes also suggested that convergent microevolution and adaption of P. aeruginosa occur in inpatients, and genomic diversity is associated with differences in multiple-drug-resistance profiles. Taken together, our findings highlight the importance of longitudinal surveillance of nosocomial P. aeruginosa clones.

## INTRODUCTION

Pseudomonas aeruginosa is a Gram-negative bacterium that is commonly found in soil and water and can also colonize humans and animals. In health care settings, P. aeruginosa can be transmitted to patients through unhygienic practices, such as the use of contaminated medical equipment, water taps, or improper hand hygiene among health care workers (1–5). Nosocomial P. aeruginosa infections are especially endemic in intensive care units (ICUs) and may involve respiratory and urinary tract, wound, and bloodstream infections. Intubated and immunocompromised patients are especially vulnerable to this common pathogen (6, 7). When a prospective multicenter study of P. aeruginosa infections in ICUs was conducted in France (the DYNAPYO cohort), 386 out of 1,561 (24.73%) patients who were not infected with P. aeruginosa at admission were found to have acquired the pathogen during their hospitalization (8). In addition, a Canadian surveillance study of over 8,000 ICU patients between 2007 and 2016 (CANWARD surveillance study) reported that P. aeruginosa accounted for 10.6% of all isolates, thereby ranking it second among microbial taxa (9).

Nosocomial infections by P. aeruginosa are associated with increased morbidity and mortality (7, 9, 10). Surveillance studies of P. aeruginosa in ICUs have also observed an increasing trend in multidrug resistance rates (9, 11–15), which may reduce the efficacy of antibiotic treatment. Multiple mechanisms, such as the overexpression of the MexXY efflux pump and a biofilm lifestyle, contribute to antibiotic resistance and adaption in P. aeruginosa. The accumulation of spontaneous mutations has also been identified as the main driver of increasing resistance, which has been demonstrated by large genomic studies of longitudinal isolates from patients with cystic fibrosis (CF) (16–19) and chronic obstructive pulmonary disease (COPD) (20, 21).

However, genomic characteristics of longitudinal P. aeruginosa isolates identified in health care settings have rarely been reported and remain largely uncharacterized. Here, we report a whole-genome analysis of 71 sequential P. aeruginosa isolates obtained from seven patients receiving ICU care for up to 19 months. Core-genome phylogeny, intraclonal genomic diversity, hypermutable isolates, and recurrently mutated genes are described.

## RESULTS

### Patient characteristics and bacterial isolates.

**FIG 1:**
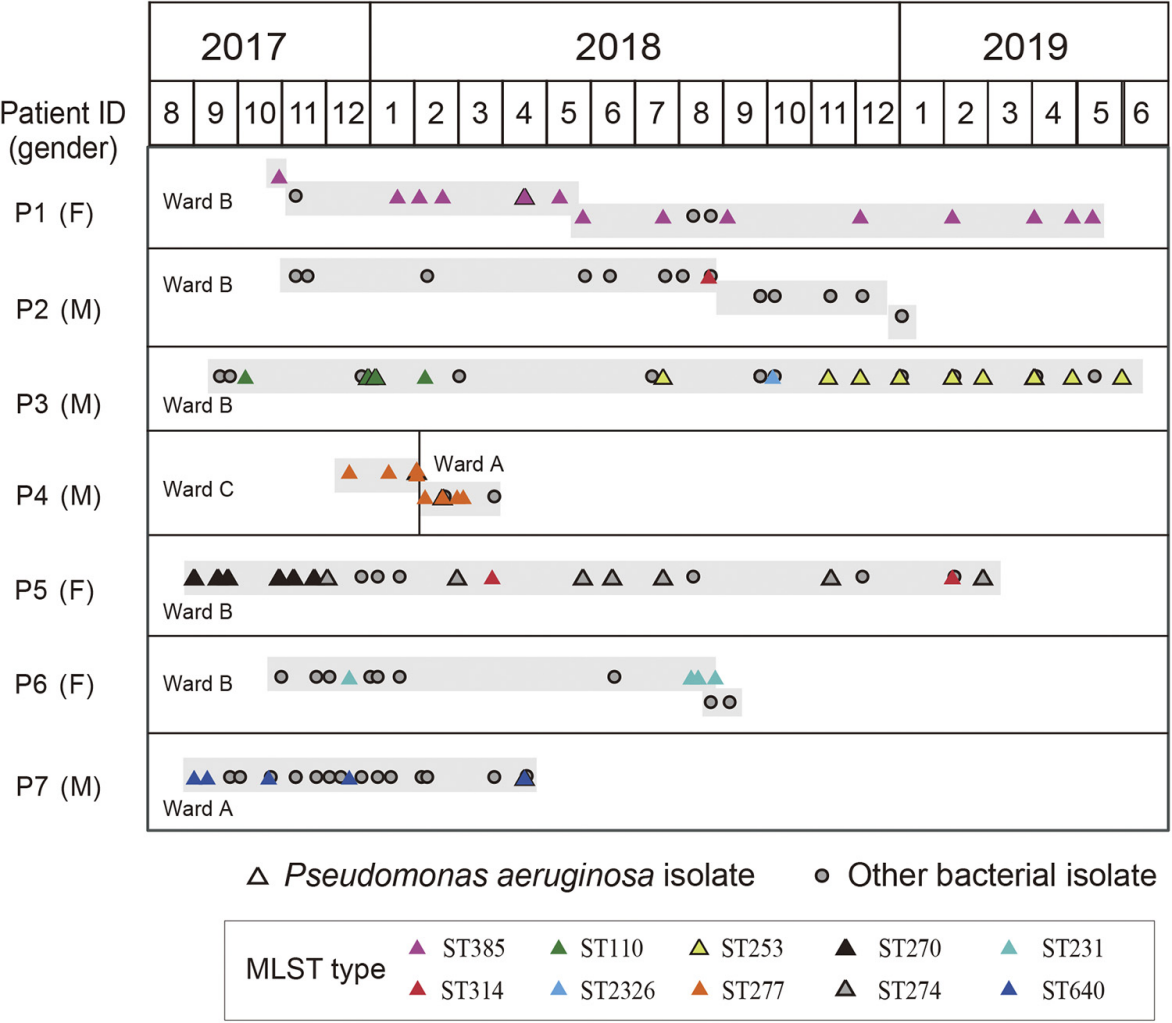
Timeline of sample collection from seven aged patients during prolonged ICU stays. Sequential samples are shown as triangles (P. aeruginosa cultivation positive) and dots (cultivation of non-P. aeruginosa organisms) according to their collection dates. The light-gray shadow indicates that the samples were collected when patients were in the same bed. Samples of P. aeruginosa are colored according to multilocus sequence typing (MLST). Details of clinical specimen types, diagnostics, and bacterial cultivations are presented in Table S1 in the supplemental material. F, female; M, male.

From August 2017 to June 2019, seven patients aged between 71 and 89 years (indexed as patients P1 to P7) were hospitalized continuously for durations ranging from 4 to 19 months in the ICUs of the 316th Hospital (Beijing, China). Six patients received continuous care in the same wards (5 patients in ward B and 1 in ward A), and patient P4 was transferred between different wards once. Six patients, except patient P6, were diagnosed with cerebral infarction and clinical manifestations of pneumonia and/or bronchitis during their hospitalization. In contrast, patient P6 had type 2 diabetes and coronary heart disease, without a medical record of infection of the lung or bronchus. A total of 139 longitudinal clinical samples containing 103 sputum specimens were collected from the seven patients for routine bacterial cultures between 2017 and 2019. Approximately half (*n* = 71) of the samples yielded P. aeruginosa, indicating that P. aeruginosa colonization or infection was prevalent and persistent. Except for only one P. aeruginosa isolate that was collected from patient P2, the durations of P. aeruginosa colonization or infection of the other six patients ranged from 3 to 20 months (Fig. 1). Detailed information regarding sample collection and bacterial isolation is provided in Table S1 in the supplemental material.

### Phylogenetic relationship of P. aeruginosa isolates.

**FIG 2:**
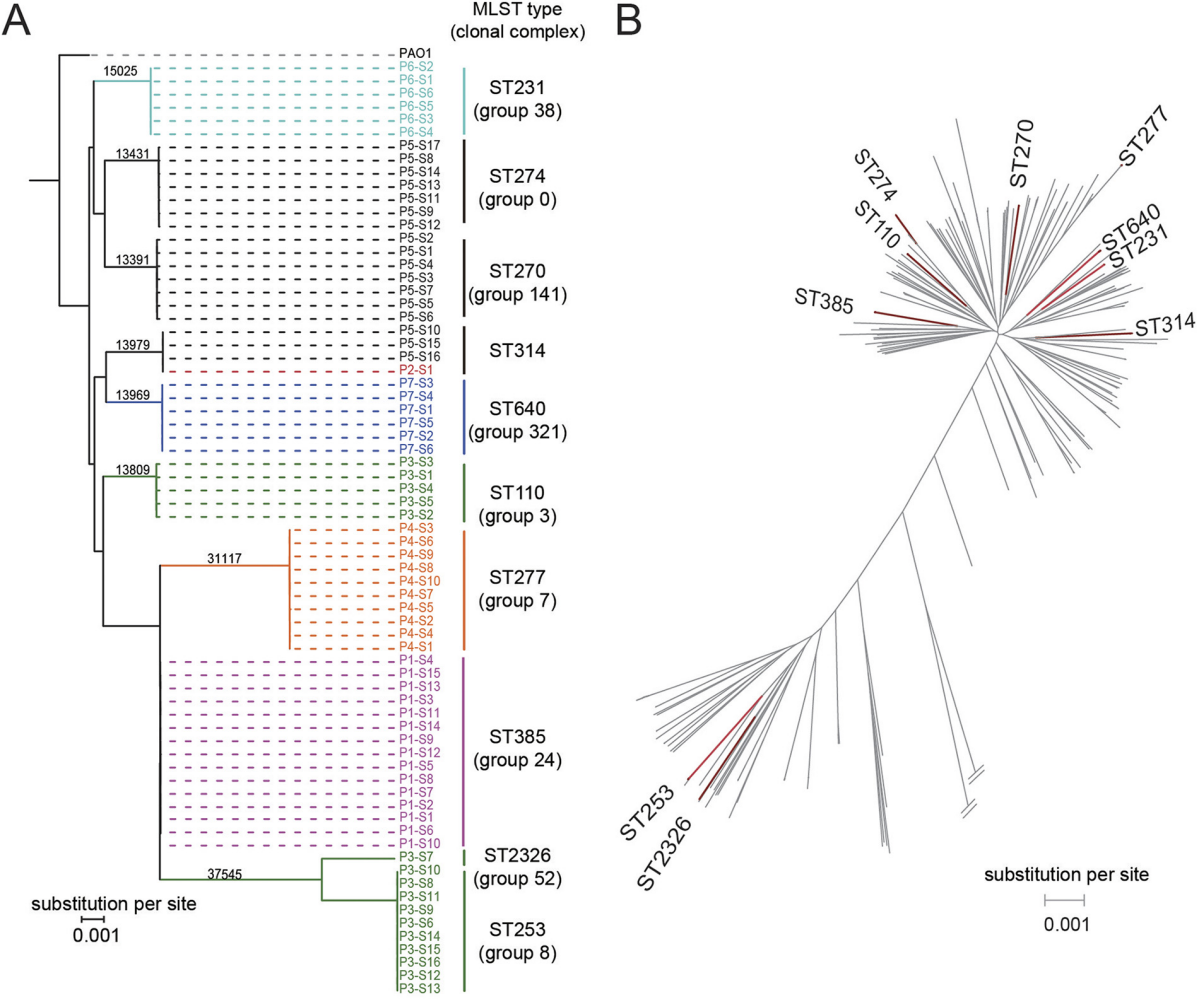
Phylogenetic relationships of P. aeruginosa isolates and switching of P. aeruginosa clones in two patients. (A) Maximum likelihood (ML) phylogenetic tree with 1,000 bootstrap replicates of the 71 isolates and the reference PAO1 strain. Branches are colored according to different patients. Multilocus sequence typing (MLST) and clonal complex identification (identified by group number) of the isolates were performed using goeBURST and are shown on the right correspondingly. The number of SNPs supporting each ST cluster is labeled on the branches. (B) ML phylogenetic tree with 1,000 bootstrap replicates based on the 71 isolates (red branches) and 357 publicly available P. aeruginosa strains (gray branches).

A phylogenetic tree revealed that the 71 P. aeruginosa isolates clustered into 10 lineages (Fig. 2A) corresponding to 10 different sequence types (STs), which also belonged to different clonal complexes (Fig. S1). Phylogenetic analysis of our isolates and 357 publicly available P. aeruginosa strains further demonstrated that the 10 STs had large phylogenetic distances (Fig. 2B).

P. aeruginosa isolates from five patients were assigned a single ST, while isolates from patients P3 and P5 had three STs. There was only one ST shared among different patients. Briefly, the P. aeruginosa isolate from patient P2 (P2-S1, collected in August 2018) was identified as belonging to ST314, identical to the transient isolates obtained from patient P5 (P5-S9, P5-S15, and P5-S16). Patient P2 had 19 sequential samples collected over 1 year, and notably, the only P. aeruginosa-positive sample, P2-S1, was from a wound drainage site. In contrast, ST314 isolates from patient P5 were from sputum and oropharyngeal swabs. Patients P2 and P5 had a temporal overlap in ward B. However, we did not find other patients who were concurrently hospitalized in ward B (patients P1, P3, and P6) and were colonized or infected with ST314 isolates.

### Clone turnover in patients.

**FIG 3:**
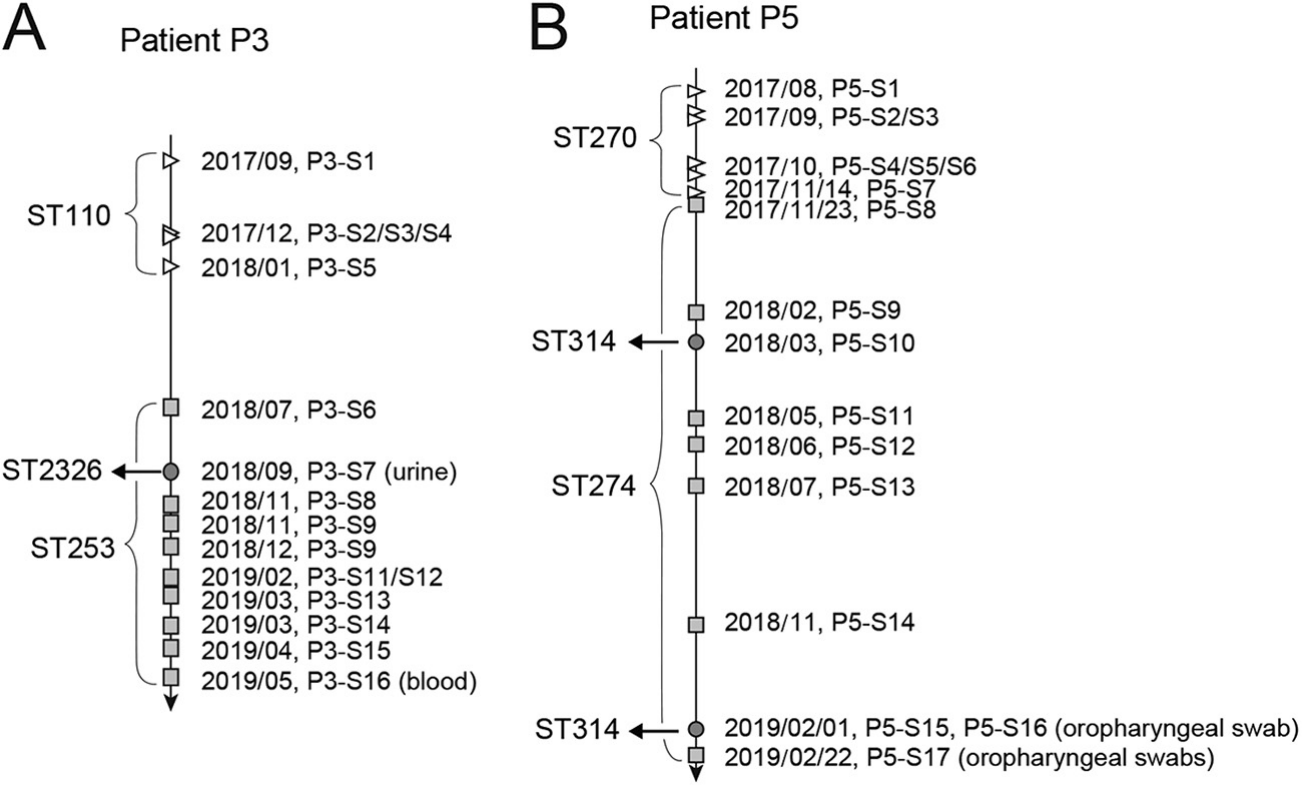
Timeline of switching of colonizing or infecting P. aeruginosa isolates in two patients. Isolates of different MLST types of patients P3 (A) and P5 (B) are shown with different shapes. Specimen types other than sputum are indicated.

Patients P3 and P5 were colonized or infected with three STs of P. aeruginosa isolates and generally exhibited two stages of colonization (Fig. 3A and B). As illustrated in Fig. 3A, patient P3 was colonized or infected with ST110 P. aeruginosa between September 2017 and January 2018, whereas isolates from sputum samples collected between July 2018 and May 2019 belonged to a different sequence type, ST253. During the process of ST253 colonization or infection, one ST2326 isolate from a urine sample was observed in September 2018.

Similar to patient P3, the sequence type of P. aeruginosa in patient P5 switched from ST270 to ST274 in November 2017. In addition, transient isolates assigned to ST314 were observed in March 2018 (P5-S9) and February 2019 (P5-S15 and P5-S16) (Fig. 3B).

### Identification of hypermutable P. aeruginosa isolates.

**FIG 4:**
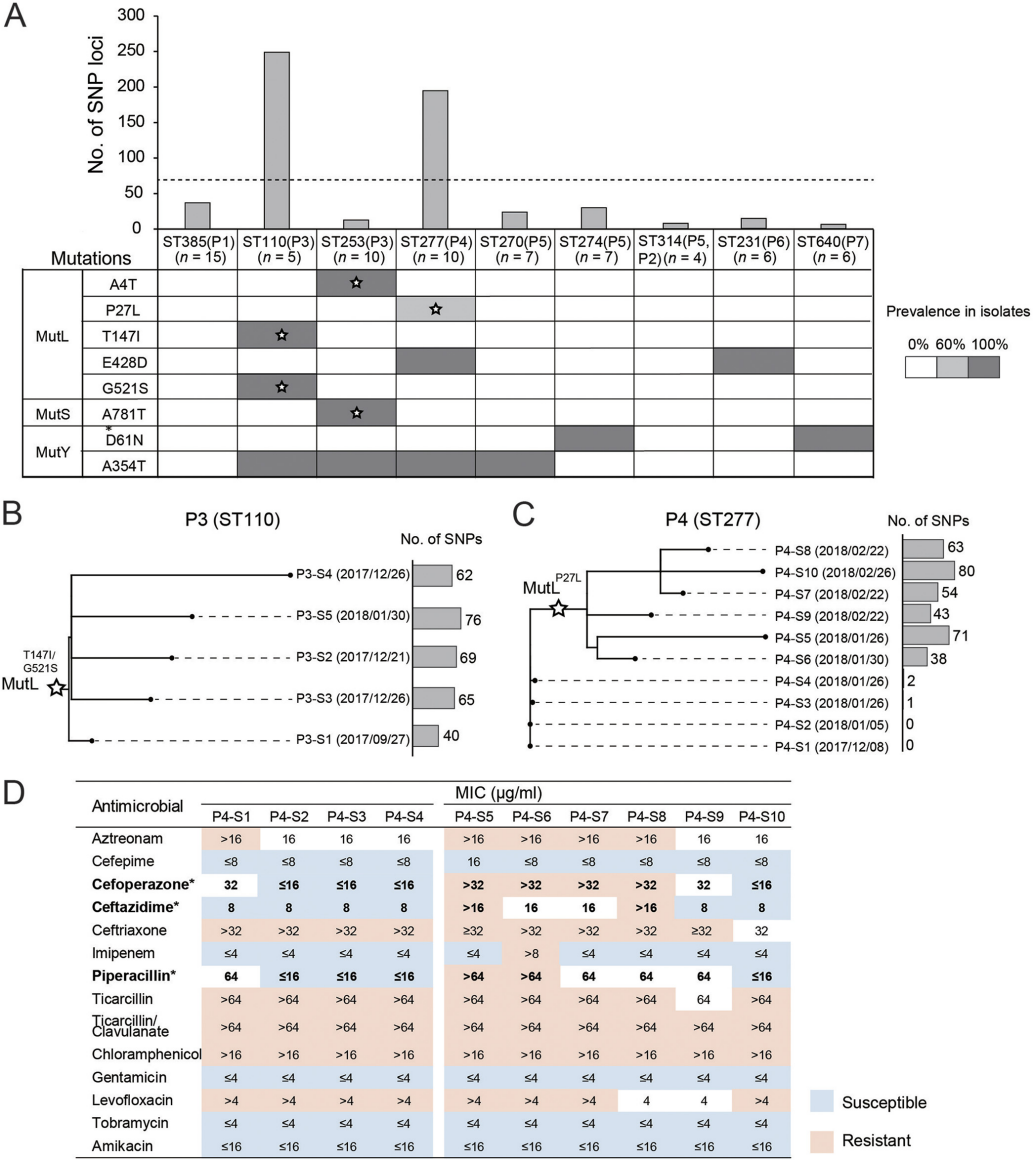
Higher numbers of mutations and phylogenies of hypermutable isolates. (A) Numbers of within-lineage SNP loci in P. aeruginosa isolates of each ST. The dashed line indicates the mean number. Mutations in the MutL, MutS, and MutY proteins in strains of each ST are shown below and indicated with gray shading. The gray shading also indicates the prevalence of mutations among isolates of each ST. Stars denote that a mutation is specific for the corresponding P. aeruginosa clones. (B and C) Maximum likelihood phylogenetic relationship of ST110 (B) and ST277 (C) hypermutable isolates. Clone-specific mutations in MutL are indicated with stars at the corresponding branches. The sample collection times and numbers of SNPs of the isolates are shown next to the tips of the phylogenetic tree. SNPs were defined according to the reference genome of PAO1. (D) Antibiotic susceptibilities of ST277 isolates from patient P4. Nonhypermutator isolates are P4-S1 to P4-S4; hypermutator isolates are P4-S5 to P4-S10. Asterisks indicate a statistically significant difference (*, *P < *0.05) between nonhypermutators and hypermutators as determined by Wilcoxon’s rank sum test.

Single nucleotide polymorphisms (SNPs) among sequential P. aeruginosa isolates with identical STs were investigated to identify hypermutable isolates. After disregarding the shared loci, loci with SNPs were analyzed for given ST isolates. The ST110 isolates from patient P3 and the ST277 isolates from patient P4 exhibited markedly higher within-lineage diversity than the isolates of other STs (Fig. S2). For example, in total, 249 and 195 SNPs were identified among the ST110 and ST277 isolates, respectively, compared with 6 to 37 SNP loci present in isolates of other STs (Fig. 4A). Moreover, the ST110 and ST277 isolates were collected over comparably short periods (4 months for ST110 and 3 months for ST277), and the corresponding numbers of isolates were moderate (*n *= 10 for ST277) or low (*n *= 5 for ST110).

The hypermutator phenotype of P. aeruginosa has been attributed to mutations in DNA mismatch repair (MMR) genes (16, 22). We identified eight nonsynonymous SNPs in the MMR genes *mutL* (*n *= 5), *mutS* (*n *= 1), and *mutY* (*n *= 2) (Fig. 4A). Amino acid polymorphisms (T147I and G521S) in protein MutL were identified in all ST110 isolates, while P27L in MutL was shared among only six ST277 isolates.

Genotypes at the within-lineage SNP loci were then obtained, and a maximum parsimonious phylogenetic model was applied to elucidate the relationships between the genotypes. The ST110 isolates, all with mutations T147I and G521S in MutL, showed remarkable intraclonal diversity. Each of the ST110 isolates contained comparable SNPs (Fig. 4B), while 90.8% (226/249) of the SNP loci were specific for each isolate. For ST277 isolates, the four isolates without P27L in MutL had highly similar genotypes and had only three SNPs, whereas 98.5% (192/195) of the within-lineage SNP loci were obtained from the six isolates bearing the P27L mutation. The majority of SNPs (72.4%; 141/192 loci) occurred once, although three of the six P27L-bearing isolates (P4-S7, P4-S8, and P4-S7) appeared to have descended from a recent common ancestor (Fig. 4C). When the antibiotic susceptibilities of the L27-bearing ST277 isolates (the hypermutators) were compared with those of their nonhypermutator counterparts, the levels of resistance to cefoperazone, ceftazidime, and piperacillin of the ST277 hypermutators were significantly higher than those of the nonhypermutators (*P* values of 0.028, 0.044, and 0.044, respectively, by a Wilcoxon rank sum test) (Fig. 4D).

**FIG 5:**
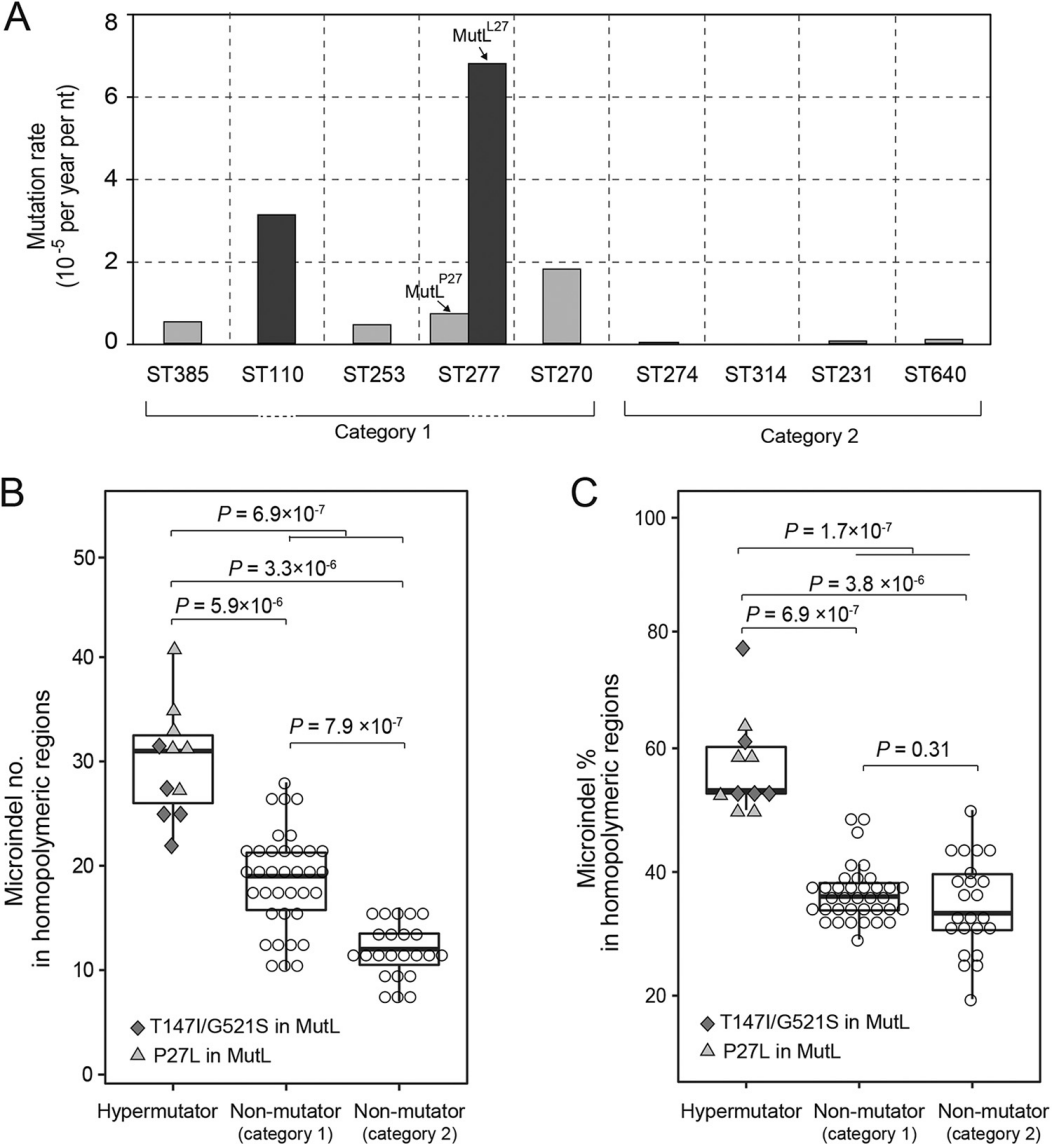
Mutation rates and microindels of hypermutable isolates and other isolates. (A) Mutation rates of P. aeruginosa isolates of different STs. The rates of mutator ST277 isolates (with L27 in MutL) and nonmutator ST277 isolates (with P27 in MutL) are shown separately. nt, nucleotide. (B and C) Isolates with P27L or T147I/G521S mutations in MutL and nonmutators are indicated by different shapes of markers. Rates for the nonmutator isolates are shown in two categories as indicated in panel A. *P* values determined by a two-sided Wilcoxon rank sum test in terms of numbers (B) and percentages (C) of microindels are shown. For the box plots, boxes represent the interquartile ranges (IQRs) between the first and third quartiles. Horizontal black lines inside the boxes indicate the median values, while the lines outside the boxes represent values within 1.5 times the IQR.

Next, the intraclonal evolutionary rates were inferred from data sets of sequential P. aeruginosa isolates by the regression of root-to-tip distances (Fig. 5A). Rates of substitution of the hypermutable ST110 isolates and L27-bearing ST277 isolates were estimated to be 3.20 × 10^−5^ and 6.59 × 10^−5^ per year per nucleotide, respectively. The other isolates could be divided into two categories. The first category contained ST314, ST274, ST231, and ST640 isolates with a median mutation rate of 3.62 × 10^−7^ (range, 0 × 10^−7^ to 9.06 × 10^−7^). The latter is approximately 2 orders of magnitude lower than those of the hypermutators. Meanwhile, the second category, which included ST385, ST253, P27-bearing ST277, and ST270 isolates, had a median mutation rate of 6.55 × 10^−6^ (range, 4.55 × 10^−6^ to 1.77 × 10^−5^), which is 1 order of magnitude lower. A mutation, A354T, in MutY was present in the isolates from the second category, except for those of ST385, and was absent in the isolates from the first category.

It has been reported that MMR-deficient P. aeruginosa strains are more prone to mutation events in homopolymeric regions than MMR-intact ones (23). Consequently, micro-insertion and deletion (microindel) events in homopolymeric regions were then identified for a comparison between hypermutators (those with T147I/G521S or P27L mutations in MutL) and other nonhypermutators. The results showed that the hypermutators had a significantly higher number of microindels in homopolymeric regions in terms of both numbers and percentages (*P* value of <7 × 10^−7^ by a two-sided Wilcoxon rank sum test) (Fig. 5B and C). Specifically, ~50.0 to 76.2% of the microindels of the hypermutators were located in homopolymeric regions, compared to ~19.4 to 50.0% for the nonhypermutators. Furthermore, the P27L-bearing isolates exhibited a slightly higher number of microindels in homopolymeric regions than did the T147I/G521S-bearing ones (Fig. 5B), yet the percentages of microindels of the two hypermutators were comparable (Fig. 5C). For the nonhypermutator isolates, the two above-mentioned groups had significantly different numbers of microindels in homopolymeric regions (Fig. 5B), yet their percentages were close (Fig. 5C).

### Recurrently mutated genes.

To investigate the existence of recurrently mutated genes from isolates of different STs, SNPs of the sequential P. aeruginosa isolates were identified using the PAO1 strain as a reference genome. Briefly, we first identified pairwise SNPs between each isolate and the PAO1 strain. SNPs shared by all of the isolates from a given ST were discarded. The remaining SNPs exhibiting diversity within the same ST were then used to define the mutations (or altered alleles) considered to be recurrently mutated genes. As a result, a total of 572 recurrent mutations were identified in isolates of the nine STs. Among them, 385 (67.3%) of the mutations were annotated as nonsynonymous (including 16 stop-gain and loss mutations), while 128 (22.4%) and 59 (10.3%) were synonymous and noncoding mutations, respectively. These mutations were distributed among 513 genes, and 24 genes were found to be recurrently mutated across the STs. Four of the 24 genes were recurrently mutated across three or more STs, and 20 genes were mutated in two STs (Fig. S3 and Table S3). A higher number of nonsynonymous mutations (67/79; 84.8%) were present in the 24 recurrently mutated genes than in the overall genome (*P* = 0.011 by two-sided Fisher’s exact test), especially among the above-mentioned 4 recurrently mutated genes in three or more STs (17 of 18; 94.4%).

Six of the 24 genes with recurrently mutated products were functionally classified as encoding transcriptional regulators (*pvdS*, *lasR*, *mexZ*, *mexS*, *fleQ*, and *PA315*), while 4 additional genes were functionally classified as encoding transporters of small molecules (*fptA*, *oprD*, *fruK*, and *PA2314*). FptA, an outer membrane ion transporter that acts via a pyochelin siderophore (24, 25), was affected by the highest number of mutations among the four STs. PvdS, a positive transcriptional regulator required for pyoverdine synthesis (26, 27), was also mutated in four STs. One particular recurrent mutation, S698F (c.2093C>T), was detected in FptA and simultaneously occurred in four different STs. A search of this mutation in the National Center for Biotechnology Information (NCBI) nucleotide database indicated that S698F in FptA has also emerged in the genomes of two P. aeruginosa strains isolated from the United States (strain DVT419 [GenBank accession number NZ_CP050328.1]) and the United Kingdom (strain T2101 [GenBank accession number NZ_CP039991.1]).

Two mutations were detected in the *oprD* gene, and both were stop-gain variants (W277* in hypermutator isolate P3-S4 and W339* in four later-stage sequential isolates of ST385 [P1-S11, P1-S13, P1-S14, and P1-S15]). Deficiency of the OprD outer membrane protein has been shown to cause β-lactam resistance (28). W277* in OprD was also previously identified in P. aeruginosa strain IRP41, which was isolated in Tianjin, China (GenBank accession number MT293231.1), and led to reduced susceptibility to carbapenems (29). Details regarding the mutations and mutation-bearing isolates are presented in Table S3 in the supplemental material.

### Structural variations in sequential isolates.

The occurrence of structural variations (SVs) was also explored in the sequential P. aeruginosa isolates. After excluding SVs shared by all isolates of the same ST, seven deletions were identified within isolates of five STs (Table S4). The lengths of these deletions ranged from 53 bp to 33.2 kbp. Two deletions were in isolates of ST385 collected from patient P1, and the other four deletions were among isolates from patients P4, P5, P6, and P7.

**FIG 6:**
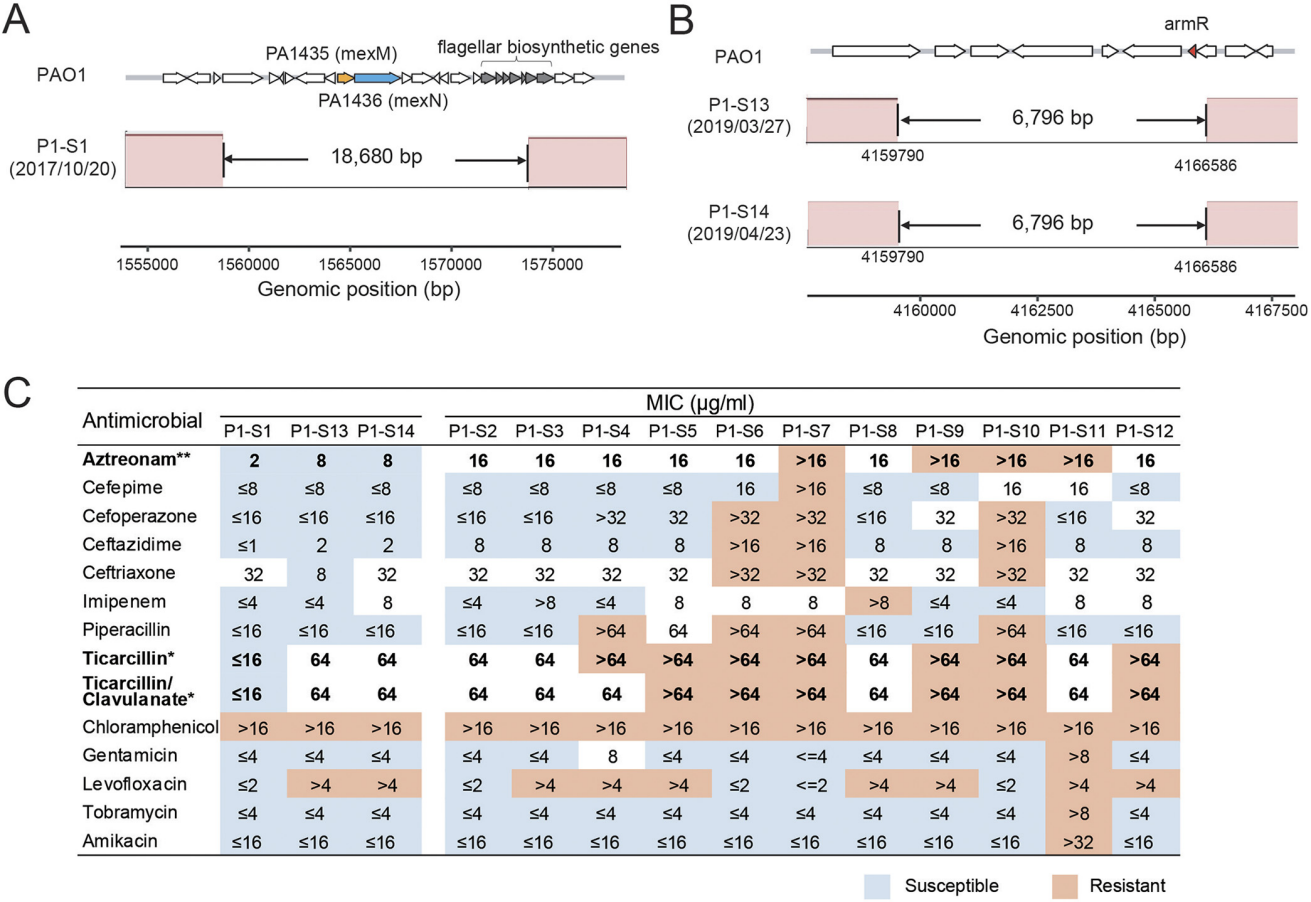
Two large deletions harboring antibiotic resistance genes. (A and B) Multiple alignments of contigs without two large deletions were obtained with the Mauve program (58). Genetic arrangements of the two regions in the PAO1 strain are shown at the top (R package gggene [https://CRAN.R-project.org/package=gggenes]) with genes involved in antibiotic resistance and flagellar assembly (*fliM*, *fliN*, *fliO*, *fliP*, *fliQ*, *fliR*, and *flhB*). The amino acid sequences of PA1435 and PA1436 in the PAO1 strain presented 98.70% and 100% identities to MexM and MexN of the PAK strain, respectively. (C) Antibiotic susceptibilities of isolates from patient P1. Isolates with deletions (P1-S1, P1-S13, and P1-S14) are shown on the left. Asterisks indicate a statistically significant difference (*, *P < *0.05; **, *P < *0.01) between isolates with the large deletions and other isolates from patient P1 as determined by Wilcoxon’s rank sum test.

Two deletions that occurred in the ST385 isolates harbored PA1435/PA1436 (highly homologous to *mexM* and *mexN* of the PAK strain) and the *armR* antibiotic resistance gene (Fig. 6A and B). Long-read sequencing further confirmed the two deletions (Fig. S4). However, both deletions were transient during the 20-month period of P. aeruginosa colonization or infection of patient P1. The deletion that included PA1435/PA1436 emerged in isolate P1-S1, collected in October 2017, while the deletion involving *armR* was identified in P1-S13 and P1-S14, collected in March and April 2019, respectively. The protein products of the *mexM* and *mexN* genes are components of the MexMN-OprM multidrug efflux pump (30), which is indirectly regulated by the ArmR protein. Isolates P1-S1, P1-P13, and P1-S14 exhibited lower levels of resistance to aztreonam, ticarcillin, and ticarcillin-clavulanate than the other isolates from patient P1 (*P* values of 0.005, 0.026, and 0.037, respectively, by a Wilcoxon rank sum test) (Fig. 6C).

The deletion in P1-S1 also included multiple flagellar assembly genes (*fliM*, *fliN*, *fliO*, *fliP*, *fliQ*, *fliR*, and *flhB*) (Fig. 6A). Similarly, the 53-bp deletion identified in five of the six ST231 isolates from patient P6 (P6-S2 to P6-S6) was also found to be related to flagellar assembly and can lead to a gene fusion of *fliR* and *fliQ* (Table S4). In contrast to the other deletions that were transient among these sequential samples, the 53-bp deletion appeared to be fixed in the clonal complex.

## DISCUSSION

Sequential isolates of P. aeruginosa were obtained from patients with prolonged ICU stays ranging from 4 to 19 months. While many studies have investigated the epidemiology of nosocomial P. aeruginosa outbreaks (5), these sequential samples provided a unique opportunity to explore the genetic diversity, evolutionary dynamics, and within-host adaption of P. aeruginosa in patients during extended ICU hospitalizations.

During their protracted ICU stays, two patients (P3 and P5) experienced clone turnovers (P3, 19 months; P5, 17 months). The infection or colonization of both patients could be divided into two stages with different dominating clones. To the best of our knowledge, this is the first report of P. aeruginosa clone turnover in ICU patients. For patient P5, the consecutive isolates defining the event of the switch from ST270 to ST274 were collected in an only 10-day interval (Fig. 3B). These data suggest that the turnover of dominating P. aeruginosa clones in patient P5 was a relatively rapid and thorough process. In contrast, the time gap between the disappearance of the ST110 clones and the emergence of the ST253 clones in patient P3 was 5.5 months (Fig. 3A). Moreover, other bacteria (Enterococcus faecalis and Enterobacter cloacae) were isolated from sputum specimens of patient P3 (see Table S1 in the supplemental material) during this period. We speculate that the new clone may have invaded a P. aeruginosa-free niche in patient P5 instead of purging a preexisting clone as in patient P3. Switches of P. aeruginosa clones have been reported in previous studies of chronic infections in patients with COPD (20) and CF (17, 31). However, sample collection in those studies extended over a much longer period (e.g., over 19 years in reference 31). The reasons for clonal turnover are interesting but rarely discussed. If longitudinal studies of P. aeruginosa in ICU patients were conducted on a fine time scale, greater insights might be obtained to address the details of clonal turnover.

Both patients P3 and P5 were colonized or infected with a third clone of P. aeruginosa. A urine sample from patient P3 yielded P. aeruginosa ST2326, indicating the coexistence of separate clones at different anatomic sites. In contrast, clones isolated from urine and sputum specimens of patient P6 were identical, and the ST314 clone in patient P5 was transient and appeared twice over an 11-month interval in respiratory tract samples. Notably, the ST314 P. aeruginosa clone was isolated from both patients P5 and P2, while all of the other clones were patient specific. Moreover, the ST314 isolates from patients P2 and P5 had only seven SNP loci, indicating that ST314 colonization or infection may have occurred as a result of indirect transmission via exposure to the same reservoir.

When sequential isolates of different clones were compared, hypermutable P. aeruginosa isolates were identified and exhibited elevated levels of both intraclonal diversity and microindels in homopolymeric regions. In addition, while eight nonsynonymous SNPs were observed in MMR genes, three SNPs (leading T147I/G521S and P27L) in MutL were found to be specifically associated with the hypermutator phenotype. All ST110 isolates harbored T147I/G521S mutations in MutL, and their nonhierarchical phylogenetic relationships (Fig. 4B) suggested that patient P3 may have acquired P. aeruginosa due to a hypermutator clone. Meanwhile, P27L in MutL could be explained in two ways. First, it was a newly emerging mutation after the colonization of patient P4 by ST277 clones. In this scenario, the effect of P27L on hypermutations seems more significant than that of T147I/G521S in MutL since it generated an ~2-fold mutation rate compared with that of the T147I/G521S-bearing ST110 hypermutators. Second, P27L and the ST277 hypermutator already existed in the clonal complex prior to colonization, and later identification of the hypermutator was due to randomness or sampling bias of the within-host heterogeneous population of P. aeruginosa.

In studies of P. aeruginosa infections in CF patients, the occurrence of hypermutable P. aeruginosa is associated with oxidative stress caused by chronic lung inflammation, and the process takes more than 5 years (32). In the present study, patient P4 may have experienced an occurrence of the hypermutators approximately 2 months after onset, and this would represent a much higher rate than that observed in CF patients. It is noteworthy that the occurrence of hypermutators in patient P4 is consistent with changes in the patient’s clinical manifestations (from “severe head injury” to “lung infection”) (Table S1). However, 5 of 7 patients in this study had lung infection or “pneumonia,” and the association between lung inflammation and the hypermutator phenotype in patient P4 may be just a coincidence.

Hypermutability can lead to a high number of deleterious mutations in a bacterial genome, and this does not confer an advantage for adaption. However, the emergence of rare and highly beneficial mutations can be accompanied by strongly linked nonadaptive variations (33), and this occurrence is referred to as “genetic draft” (34, 35). Among the 24 recurrently mutated genes identified in our study, 14 and 15 of these mutated genes were identified in hypermutable ST110 and ST277 isolates, respectively (Table S3). Many of the recurrently mutated genes were found to be related to the adaptation of P. aeruginosa to the host environment. However, the ST110 hypermutable strain disappeared in patient P3 after 5 months. Clearance of hypermutable isolates has not been typically observed for P. aeruginosa infections in CF and COPD patients (36). On the other hand, the other hypermutator, the ST277 isolate with the P27L mutation in MutL in patient P4, was observed for less than a month. Further studies are still needed to determine how hypermutability contributes to the adaption of P. aeruginosa in acute nosocomial infections and whether it differs from that in chronic infections.

We considered recurrent mutations as a readout of convergent microevolution since important functional mutations are more likely to cooccur in independent clones. In general, the most prominent recurrently mutated genes exhibited high concordance with known characteristics of convergent evolution and the adaptation of P. aeruginosa in CF patients (16, 17, 31). For example, P. aeruginosa increases its metal ion acquisition function after its growth environment changes from soil and water to the human respiratory tract, where essential nutrients such as iron and zinc are limited (26, 27). The two most recurrently mutated proteins in our study were FptA and PvdS, which contribute to pyochelin and pyoverdine synthesis. S698F in FptA was notable as it was the only recurrent mutation emerging in four clones and other P. aeruginosa strains (DVT419 and T2101). In CF patients, remodeling of regulatory networks is important for the adaptation of P. aeruginosa. In addition to the transcriptional regulator PvdL, many of the recurrently mutated proteins, including MexZ, LasR, FleQ, and MexS, are all transcriptional regulators that play important roles in antibiotic resistance and biofilm formation. For example, MexZ is a negative regulator that directly controls the expression of the MexXY efflux pump, and mutations in MexZ increase pump expression and antibiotic resistance (16). LasR is a transcriptional activator of quorum sensing, which is closely interconnected to biofilm development (37–39). Meanwhile, MexS regulates the multidrug efflux pump MexEF-OprN, and its deficiency leads to multidrug resistance (40–42). We also identified gene dysfunction related to flagellar assembly as a result of large deletion mutations in two clones (ST385 and ST231). Flagellar loss contributes to a pathophenotype that emerges after P. aeruginosa switches to an aggregative lifestyle (16). The recurrently mutated genes indicate convergent within-host microevolution, which is beneficial for the adaption of P. aeruginosa to the host environment. Previous studies have indicated that most antibiotic resistance mechanisms were associated with reduced bacterial growth rates, but the effects of these mutations on the fitness of P. aeruginosa still need further investigation (43, 44).

In summary, our study of the genomic dynamics of P. aeruginosa in ICU patients, including clonal turnover and the emergence of a novel hypermutator phenotype, emphasizes the importance of longitudinal surveillance of nosocomial transmission and infections.

## MATERIALS AND METHODS

### Ethics statement.

This study was approved and supervised by the Institutional Review Board of the Chinese PLA Center for Disease Control and Prevention. The study was conducted in accordance with the Declaration of Helsinki, as revised in 2013. No identifiable participant data were included in this study. The ICU wards were pseudoanonymized (the letters A, B, and C were used during data collection and analysis in place of routinely used ward numbers).

### Bacterial isolation and whole-genome sequencing of P. aeruginosa.

Isolates were recovered from clinical samples of inpatients in the ICUs of the 316th Hospital, Beijing, China. The strains were grown overnight in Luria-Bertani broth (Solarbio, China) at 37°C. Species-level identification was performed using a Vitek 2 compact system (bioMérieux, France).

Genomic DNA was extracted from cultured isolates using a High Pure PCR template preparation kit (Roche, Switzerland). The sequencing library was prepared using a NEBNext DNA preparation kit (New England BioLabs, USA) according to the manufacturer’s instructions, with an insert size of about 350 bp. Whole-genome sequencing (WGS) was conducted for all P. aeruginosa isolates using an Illumina NovaSeq 6000 system (Illumina, USA) by Novogene Co., Ltd. (Beijing, China), to generate 2 × 150-bp paired-end reads (on average, 1.68 Gbp per isolate [standard deviation {SD}, 0.23 Gbp]) (see Table S1 in the supplemental material).

Seven P. aeruginosa isolates underwent long-read WGS for structural variation validation. A MinION sequencer (Oxford Nanopore Technologies, UK) was used to generate long sequencing reads with an R9.4.1 flow cell and a ligation sequencing kit (catalog number SQK-LSK109). Guppy v3.2.1 was employed for base calling using the high-accuracy model.

### Antimicrobial susceptibility testing.

The antimicrobial susceptibilities of the P. aeruginosa isolates were determined by using the Vitek 2 compact system (bioMérieux, France) according to the manufacturer’s instructions. The MICs of aztreonam, cefepime, cefoperazone, ceftazidime, ceftriaxone, imipenem, piperacillin, ticarcillin, ticarcillin-clavulanate, chloramphenicol, gentamicin, levofloxacin, tobramycin, and amikacin were determined, and the results were interpreted according to Clinical and Laboratory Standards Institute (CLSI) guidelines (45). The antibiotic susceptibilities of the P. aeruginosa isolates are provided in Table S2.

### Genome assembly and annotation.

Next-generation sequencing (NGS) reads were examined by FastQC, and *de novo* assembly was conducted using SPAdes v3.13.0 (46) with candidate *k*-mer sizes of 21, 33, 55, 77, 99, and 127. Genome annotation was performed with Prokka 1.14.6 (47), with the genome of the P. aeruginosa PAO1 strain as a reference. The core genome of P. aeruginosa was inferred using Roary v3.7.0 (48). Functional and pathway classifications of individual genes were obtained from the Pseudomonas Genome Database version 20.2 (https://www.pseudomonas.com/) (49).

### Genomic variation identification and annotation.

The identification of single nucleotide variations (SNVs) or single nucleotide polymorphisms (SNPs) was performed as described in our previous study (50). Briefly, NGS reads were aligned to a reference genome (P. aeruginosa PAO1 strain [GenBank accession number NC_002516]) with BWA mem v0.7.17 (51). SAMtools v1.9 (52) was used to generate mpileup files based on alignment with the parameters -A –B –Q 20. The sequencing bases at genomic positions were analyzed via homemade scripts (http://github.com/generality/iSNV-calling). *Q*_20_ quality filtering was used to calculate sequencing bases. Variations with a ≥50-fold depth and with a minor allele frequency of ≥0.05 were obtained. SNPs were defined as variations with a mutated allele frequency of ≥0.9.

Both VarScan v2.4.4 (53) and Pindel v0.2.5b9 (54) were implemented to identify micro-insertions and deletions (microindels). The filters for VarScan were set as a minimum sequencing depth at the locus of ≥50-fold, at least two reads supporting the allele, and an allele frequency of >0.1. The parameters for Pindel were set as a mapping quality of the aligned reads of ≥30 (-A 30), ≥10 reads supporting the microindel events (-M 10), and a 360-bp insert size. Microindels identified by both VarScan and Pindel, with sizes of <50 bp, were obtained. Microindels within homopolymeric sequences were extracted from the outputs of Pindel with the labels HOMSEQ and HOMLEN, which had a required homopolymeric length of ≥3 bp. For the detection of structural variations (SVs), we used both DELLY v0.8.7 (55) and Pindel v0.2.5b9 (54). DELLY was implemented with the default parameters. Insertions and deletions with lengths of ≥50 bp identified by both DELLY and Pindel were extracted.

SnpEff v4.3t (56) was used to annotate genomic variations based on the reference genome of the PAO1 strain. The Integrative Genomics Viewer (57) and Mauve v2.4.0 (58) were utilized for manual inspection and visualization at the read and assembly levels, respectively.

### Phylogenetic analysis.

SNPs of the core genomes of the P. aeruginosa isolates were used for phylogenetic analysis, and maximum likelihood (ML) phylogenies were inferred by RAxML v8.2.12 (59) with 1,000 bootstrap replicates using the GTR+γ model. An ascertainment bias correction of the GTR+Γ model was conducted with the parameters -m ASC_GTRGAMMA –asc-corr=stamatakis.

The phylogenetic relationships of the isolates and publicly available P. aeruginosa strains were analyzed using a core-genome-based ML method. A total of 357 complete genomes of P. aeruginosa were downloaded from the NCBI GenBank database (https://www.ncbi.nlm.nih.gov/genome/browse/#!/prokaryotes/187/) on 15 September 2021. The core genomes were obtained with Roary v3.7.0 (48). The ML phylogenies were inferred using FastTree v2.1.11 with the GTR model (60). The trees generated by RAxML and FastTree were visualized using iTOL (https://itol.embl.de/) (61). ML phylogenetic trees of different genotypes at SNP loci of isolates of the same sequence type (ST) were obtained and visualized using MEGA X (62) with a Tamura-Nei model (63).

### Multilocus sequence typing and clonal complex.

Multilocus sequence typing (MLST) was conducted based on the whole genomes of the P. aeruginosa isolates. The nucleotide sequences of the seven housekeeping genes of P. aeruginosa, *acsA*, *aroE*, *guaA*, *mutL*, *nuoD*, *ppsA*, and *trpE*, were obtained, and the ST was determined via public databases for molecular typing and microbial genome diversity (https://pubmlst.org/organisms/pseudomonas-aeruginosa/) (64).

The relationships of P. aeruginosa STs were analyzed using goeBURST v1.2.1 (http://www.phyloviz.net/goeburst/) (65), and the clonal complexes that contain each ST identified in the inpatients were obtained.

### Estimation of mutation rates.

The mutation rates of the P. aeruginosa isolates with the same ST were inferred using TempEST v1.5.3 (66). The SNP-based RAxML tree and the sampling times of the isolates were used as the input for the TempEST program. The slope of the root-to-tip regression was used to estimate the mutation rate.

### Statistical analysis.

Statistical analysis was conducted using R software v3.5.3 (R Foundation for Statistical Computing). Statistical significance between groups was calculated using a two-sided Wilcoxon rank sum test and two-sided Fisher’s exact test.

### Data availability.

The raw NGS data of P. aeruginosa reported in this study have been deposited in the NCBI Sequence Read Archive (SRA) database under BioProject accession number PRJNA729183. The genome assemblies of P. aeruginosa have been deposited at the National Genomics Data Center (NGDC) of the China National Center for Bioinformation under BioProject accession number PRJCA007119 (https://ngdc.cncb.ac.cn/). The scripts for the bioinformatics analysis in this study are available at https://github.com/Ming-Ni-Lab/P.aeruginosa-ICU-patients.
