# Supplementary material for: Time Series Genomics of Pseudomonas aeruginosa Reveals the Emergence of a Hypermutator Phenotype and Within-Host Evolution in Clinical Inpatients
Source: Microbiol Spectr. 2022 Jul 21;10(4):e00057-22. doi: 10.1128/spectrum.00057-22 (PMC9430856; doi:10.1128/spectrum.00057-22)
Supplement: Supplemental file 1 — Supplemental material. Download spectrum.00057-22-s0001.pdf, PDF file, 1.6 MB [file spectrum.00057-22-s0001.pdf]

Supplementary materials

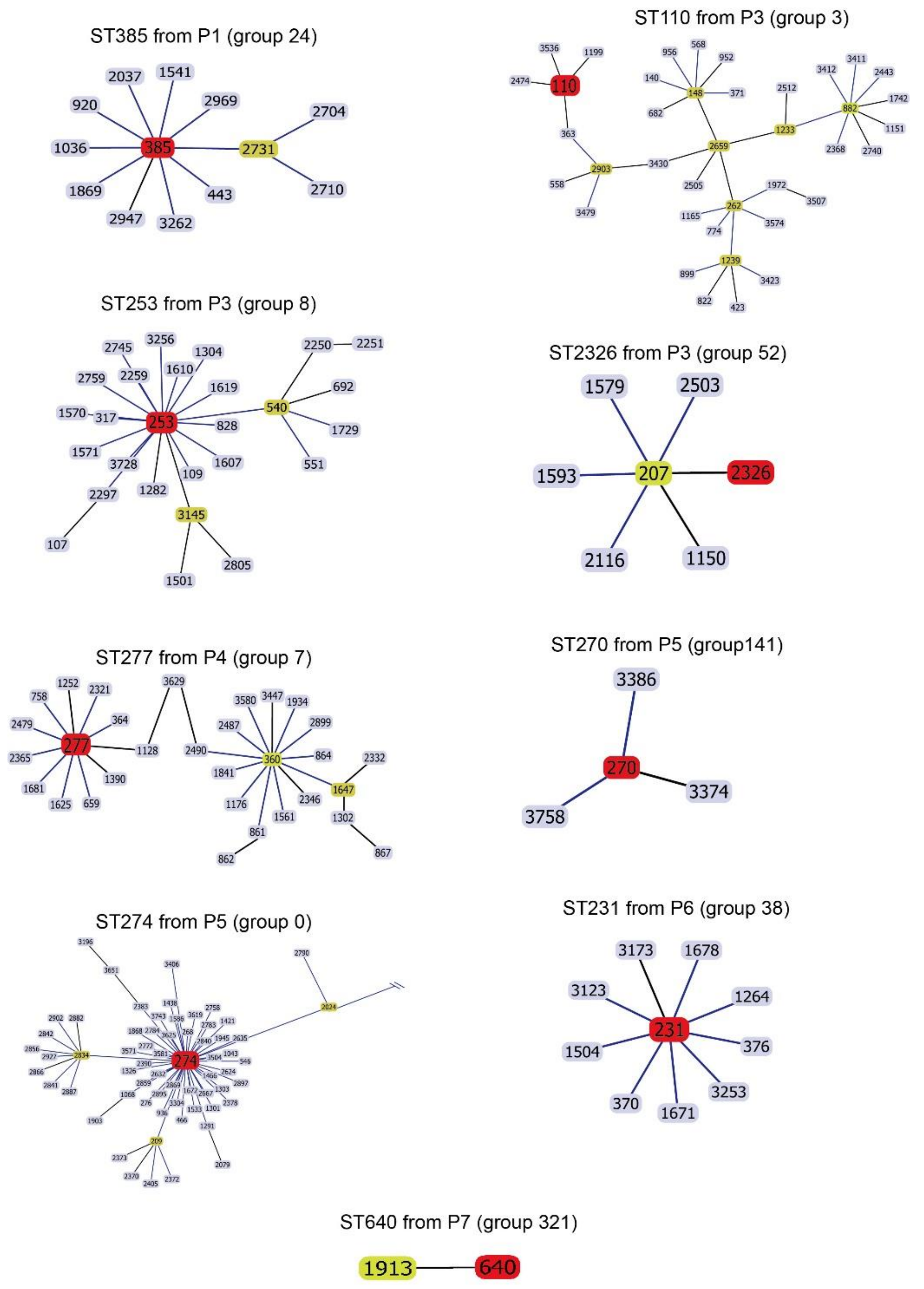

Supplementary material Figure S1. Clonal complexes of the *P. aeruginosa* isolates. The clonal complexes (groups of closely related strains) of the *P. aeruginosa* isolates were obtained and illustrated as networks by using the goeBURST v1.2.1 software (http://www.phyloviz.net/goeburst/). The MLST types identified in this study (red node in network) and the corresponding inpatients, as well as the group numbers, are indicated at the top of each network. The ST314 from inpatients P5 and P2 were not found by goeBURST and thus not shown.

ST385

n	P1-S1	P1-S2	P1-S3	P1-S4	P1-S5	P1-S6	P1-S7	P1-S8	P1-S9	P1-S10	P1-S11	P1-S12	P1-S13	P1-S14	P1-S15
P1-S1															
P1-S2	2														
P1-S3	5	3													
P1-S4	7	5	2												
P1-S5	7	5	2	4											
P1-S6	4	4	7	9	9										
P1-S7	1	1	4	6	6	3									
P1-S8	6	4	1	3	3	8	5								
P1-S9	8	6	3	5	5	10	7	4							
P1-S10	5	5	8	10	10	3	4	9	11						
P1-S11	18	16	13	15	15	20	17	14	16	21					
P1-S12	8	6	3	5	5	10	7	4	0	11	16				
P1-S13	20	18	15	17	17	22	19	16	18	23	4	18			
P1-S14	20	18	15	17	17	22	19	16	18	23	4	18	6		
P1-S15	18	16	13	15	15	20	17	14	16	21	2	16	2	4	

ST277

n	P4-S1	P4-S2	P4-S3	P4-S4	P4-S5	P4-S6	P4-S7	P4-S8	P4-S9	P4-S10
P4-S1										
P4-S2	0									
P4-S3	1	1								
P4-S4	2	2	3							
P4-S5	71	71	72	73						
P4-S6	37	37	38	39	56					
P4-S7	54	54	55	56	85	51				
P4-S8	62	62	63	64	93	59	26			
P4-S9	42	42	43	44	73	39	54	62		
P4-S10	80	80	81	82	111	77	44	52	80	

ST253

n	P3-S6	P3-S8	P3-S9	P3-S10	P3-S11	P3-S12	P3-S13	P3-S14	P3-S15	P3-S16
P3-S6										
P3-S8	4									
P3-S9	2	6								
P3-S10	1	5	3							
P3-S11	4	8	2	5						
P3-S12	4	8	6	5	8					
P3-S13	4	8	6	5	8	0				
P3-S14	4	8	6	5	8	0	0			
P3-S15	4	8	6	5	8	0	0	0		
P3-S16	4	8	6	5	8	0	0	0	0	

ST270

n	P5-S1	P5-S2	P5-S3	P5-S4	P5-S5	P5-S6	P5-S7
P5-S1							
P5-S2	1						
P5-S3	13	12					
P5-S4	13	12	0				
P5-S5	19	18	10	10			
P5-S6	19	18	10	10	0		
P5-S7	18	17	9	9	7	7	

ST274

n	P5-S8	P5-S9	P5-S11	P5-S12	P5-S13	P5-S14	P5-S17
P5-S8							
P5-S9	20						
P5-S11	21	1					
P5-S12	21	1	2				
P5-S13	22	2	3	3			
P5-S14	21	1	2	2	3		
P5-S17	9	21	22	22	23	22	

ST231

n	P6-S1	P6-S2	P6-S3	P6-S4	P6-S5	P6-S6
P6-S1						
P6-S2	11					
P6-S3	11	0				
P6-S4	11	0	0			
P6-S5	12	3	3	3		
P6-S6	10	1	1	1	2	

ST640

n	P7-S1	P7-S2	P7-S3	P7-S4	P7-S5	P7-S6
P7-S1						
P7-S2	2					
P7-S3	0	2				
P7-S4	1	3	1			
P7-S5	0	2	0	1		
P7-S6	5	3	5	6	5	

ST110

n	P3-S1	P3-S2	P3-S3	P3-S4	P3-S5
P3-S1					
P3-S2	67				
P3-S3	59	92			
P3-S4	97	124	122		
P3-S5	74	101	99	131	

ST314

n	P2-S1	P5-S10	P5-S15	P5-S16
P2-S1				
P5-S10	2			
P5-S15	3	3		
P5-S16	4	4	5	

Supplementary material Figure S2. Pair-wise genomic differences of the intra-clonal isolates. Based on the core genome, the numbers of single nucleotide polymorphisms (*n*) between *P. aeruginosa* isolates of identical MLST type were calculated. The MLST types are indicated at the top. These MLST types were divided into separate clonal complexes (see supplementary material Figure S1). ST2326 was not shown as that it had only one isolate (P3-S7 from inpatient P3).

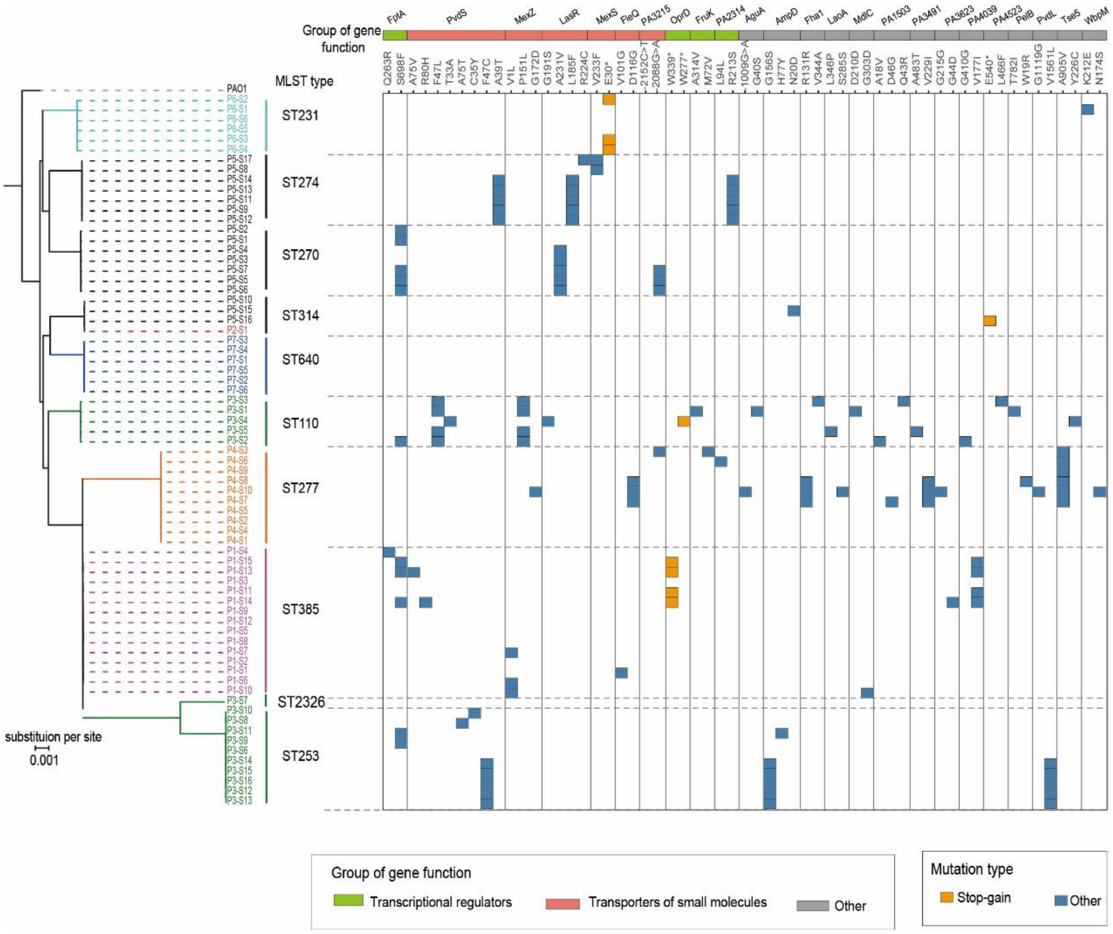

Supplementary material Figure S3. Phylogenetic tree of P. aeruginosa isolates from different MLST types and correlation with recurrently mutated genes. Isolates from different patients are distinguished by color. Multi-locus sequence typing (MLST) are shown aside correspondingly. The orange and blue boxes showed the stop-gain and other types of mutations. The groups of gene function are indicated at the top by the bar graph filled in green, red and gray.

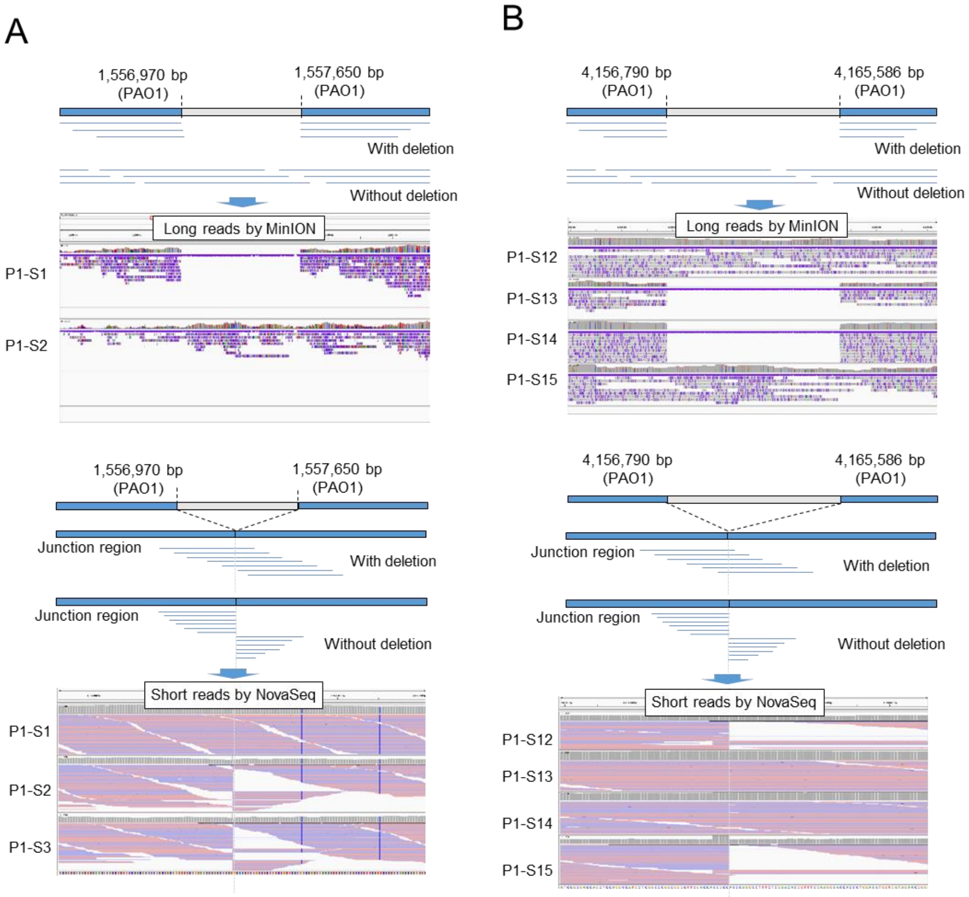

Supplementary material Figure S4. Validation of two large deletions that harbored antibiotic resistance genes. The *P. aeruginosa* isolates P1-S1 (**A**) and P1-S13/P1-S14 (**B**) were identified with deletions that included genes PA1435/PA1436 and *armR*, respectively. The deletions were validated by both using long-read sequencing (MinION sequencer, Oxford Nanopore technologies, UK) and re-alignment of next-generation sequencing short reads (NovaSeq 6000 system, Illumina, USA). The diagrams of long and short reads alignment for isolates with and without deletions are shown. The nanopore sequencing reads of the isolates harboring the deletions and their consecutive isolates were aligned to reference genome of PAO1 strain, exhibited at the upper panel. At the lower panel, the short reads were re-aligned to the sequences harboring the corresponding deletions. As the short reads could not cover the deletions, we manually linked the upstream- and downstream regions of the deletions as junction regions, and the short reads were aligned to the junction regions for illustration. The reads of P1-S1 and P1-S13/P1-S14 covered the junction sites of the deletions; whereas the consecutive isolates had soft-clipped reads for the alignments. All alignments were visualized by using the Integrative Genomics Viewer. The genomic coordinates of the deletions were indicated, with the genome of PAO1 strain as reference sequences.

Supplementary material Table S1. Patients' characteristics and culturing isolates. The samples were collected from seven elderly patients who hospitalized persistently in the intensive care unit (ICU) of the 316th hospital, Beijing, China. The raw sequencing data of the *P. aeruginosa* isolates have been deposited in NCBI SRA database under the BioProject PRJNA729183 (https://www.ncbi.nlm.nih.gov/bioproject/PRJNA729183). Genome assemblies of the 71 *P. aeruginosa* isolates are available at National Genomics Data Center (NGDC) of China National Center for Bioinformation via the link https://ngdc.cncb.ac.cn/search/?dbId=gwh&q=PRJCA007119. ID, Identifier.

Patient (gender, age)	Ward	Type of specimen	Sample collection year and month	Clinical manifestation	Identification of isolate	ID for *P. aeruginosa* isolate	MSLT type of *P. aeruginosa* isolate	BioSample accession (NCBI SRA)	Genome assembly accession (NGDC)
	B	Sputum	2017/10/20	Pneumonia	*Pseudomonas aeruginosa*	P1-S1	ST385	SAMN19 112879	GWHBFW B00000000
	B	Blood	2017/10/30	Pneumonia	*Staphylococcu s capitis*				
	B	Sputum	2018/1/11	Pneumonia, Pleural effusion	*Pseudomonas aeruginosa*	P1-S2	ST385	SAMN19 112880	GWHBFTJ0 0000000
	B	Sputum	2018/1/26	Pneumonia, Pleural effusion	*Pseudomonas aeruginosa*	P1-S3	ST385	SAMN19 112881	GWHBFTK 00000000
	B	Sputum	2018/2/12	Pneumonia, Pleural effusion	*Pseudomonas aeruginosa*	P1-S4	ST385	SAMN19 112882	GWHBFTL 00000000
	B	Sputum	2018/4/8	Pneumonia, Bronchiectasis	*Pseudomonas aeruginosa*	P1-S5	ST385	SAMN19 112883	GWHBFTM 00000000
	B	Sputum	2018/4/8	Pneumonia, Bronchiectasis	*Pseudomonas aeruginosa*	P1-S6	ST385	SAMN19 112884	GWHBFTN 00000000
	B	Sputum	2018/5/2	Cerebral infarction, Bronchiectasis	*Pseudomonas aeruginosa*	P1-S7	ST385	SAMN19 112885	GWHBFTO 00000000
P1 (Femal e,89)	B	Sputum	2018/5/18	Cerebral infarction, Bronchiectasis	*Pseudomonas aeruginosa*	P1-S8	ST385	SAMN19 112886	GWHBFTP 00000000
	B	Sputum	2018/7/13	Acute cerebral infarction	*Pseudomonas aeruginosa*	P1-S9	ST385	SAMN19 112887	GWHBFTQ 00000000
	B	Wound Secretion	2018/8/2	Acute cerebral infarction	*Klebsiella pneumoniae*				
	B	Wound Secretion	2018/8/14	Acute cerebral infarction	*Klebsiella pneumoniae*				
	B	Sputum	2018/8/27	Acute cerebral infarction	*Pseudomonas aeruginosa*	P1-S10	ST385	SAMN19 112888	GWHBFTR 00000000
	B	Sputum	2018/11/28	Acute cerebral infarction	*Pseudomonas aeruginosa*	P1-S11	ST385	SAMN19 112889	GWHBFTS 00000000
	B	Sputum	2019/2/1	Acute cerebral infarction	*Pseudomonas aeruginosa*	P1-S12	ST385	SAMN19 112890	GWHBFTT 00000000
	B	Sputum	2019/3/27	Acute cerebral infarction	*Pseudomonas aeruginosa*	P1-S13	ST385	SAMN19 112891	GWHBFTU 00000000
	B	Sputum	2019/4/23	Acute cerebral infarction	*Pseudomonas aeruginosa*	P1-S14	ST385	SAMN19 112892	GWHBFTV 00000000
	B	Sputum	2019/5/7	Acute cerebral infarction	*Pseudomonas aeruginosa*	P1-S15	ST385	SAMN19 112893	GWHBFTW 00000000
	B	Sputum	2017/10/30	acute respiratory tract infections	*Enterobacter cloacae*				
	A	Sputum	2017/11/8	Pneumonia	*Escherichia coli*				
	B	Sputum	2018/1/30	Cerebral infarction	*Escherichia coli*				
	B	Sputum	2018/5/18	Cerebral infarction	*Klebsiella pneumoniae*				
	B	Wound Secretion	2018/6/5	Cerebral infarction	*Staphylococcu s aureus*				
P2 (Male,7 7)	B	Blood	2018/7/13	Cerebral infarction	*Staphylococcu s hominis*				
	B	Wound Secretion	2018/7/13	Cerebral infarction	*Staphylococcu s haemolyticus*				
	B	Sputum	2018/7/25	Pneumonia, Cerebral infarction	*Escherichia coli*				
	B	Wound Secretion	2018/8/14	Cerebral infarction	*Pseudomonas aeruginosa*	P2-S1	ST314	SAMN19 112894	GWHBFTX 00000000
	B	Wound Secretion	2018/8/14	Cerebral infarction	*Escherichia coli*				
	B	Sputum	2018/9/18	Cerebral infarction	*Escherichia coli*				
	B	Sputum	2018/9/18	Cerebral infarction	*Escherichia coli*				
	B	Sputum	2018/9/18	Cerebral infarction	*Escherichia coli*				

	B	Sputum	2018/9/28	Acute exacerbation of chronic bronchitis	*Escherichia coli*				
	B	Sputum	2018/11/6	Acute exacerbation of chronic bronchitis	*Escherichia coli*				
	B	Sputum	2018/11/6	Acute exacerbation of chronic bronchitis	*Acinetobacter baumannii*				
	B	Sputum	2018/11/28	Acute exacerbation of chronic bronchitis	*Escherichia coli*				
	B	Blood	2018/12/25	Acute exacerbation of chronic bronchitis	*Staphylococcus epidermidis*				
	B	Sputum	2018/12/25	Acute exacerbation of chronic bronchitis	*Escherichia coli*				
P3 (male,81)	B	Sputum	2017/9/8	Pneumonia	*Pseudomonas fluorescens*				
	B	Sputum	2017/9/15	Pneumonia	*Pseudomonas fluorescens*				
	B	Sputum	2017/9/27	Pneumonia	*Pseudomonas aeruginosa*	P3-S1	ST110	SAMN19112895	GWHBFTY00000000
	B	Urine	2017/12/15	Cerebral infarction	*Enterococcus faecalis*				
	B	Sputum	2017/12/21	Cerebral infarction	*Pseudomonas aeruginosa*	P3-S2	ST110	SAMN19112896	GWHBFTZ00000000
	B	Urine	2017/12/21	Cerebral infarction	*Enterococcus durans*				
	B	Sputum	2017/12/26	Cerebral infarction	*Pseudomonas aeruginosa*	P3-S3	ST110	SAMN19112897	GWHBFUA00000000
	B	Sputum	2017/12/26	Cerebral infarction	*Pseudomonas aeruginosa*	P3-S4	ST110	SAMN19112898	GWHBFUB00000000
	B	Sputum	2018/1/30	Cerebral infarction	*Pseudomonas aeruginosa*	P3-S5	ST110	SAMN19112899	GWHBFUC00000000
	B	Sputum	2018/2/22	Cerebral infarction	*Enterococcus faecalis*				
	B	Sputum	2018/7/4	Cerebral infarction	*Enterobacter cloacae*				
	B	Sputum	2018/7/13	Cerebral infarction	*Pseudomonas aeruginosa*	P3-S6	ST253	SAMN19112900	GWHBFUD00000000
	B	Urine	2018/9/18	Cerebral infarction	*Proteus mirabilis*				
	B	Blood	2018/9/18	Cerebral infarction	*Staphylococcus hominis*				
	B	Urine	2018/9/18	Cerebral infarction	*Proteus mirabilis*				
	B	Urine	2018/9/18	Cerebral infarction	*Enterococcus faecium*				
	B	Urine	2018/9/28	Cerebral infarction	*Enterococcus faecium*				
	B	Urine	2018/9/28	Cerebral infarction	*Pseudomonas aeruginosa*	P3-S7	ST2326	SAMN19112901	GWHBFUE00000000
	B	Sputum	2018/11/6	Cerebral infarction	*Pseudomonas aeruginosa*	P3-S8	ST253	SAMN19112902	GWHBFUF00000000
	B	Sputum	2018/11/28	Cerebral infarction	*Pseudomonas aeruginosa*	P3-S9	ST253	SAMN19112903	GWHBFUG00000000
	B	Sputum	2018/12/25	Cerebral infarction	*Pseudomonas aeruginosa*	P3-S10	ST253	SAMN19112904	GWHBFUH00000000
	B	Urine	2018/12/25	Cerebral infarction	*Proteus mirabilis*				
	B	Urine	2019/2/1	Cerebral infarction	*Proteus mirabilis*				
	B	Sputum	2019/2/1	Cerebral infarction	*Pseudomonas aeruginosa*	P3-S11	ST253	SAMN19112905	GWHBFUI00000000
	B	Sputum	2019/2/22	Cerebral infarction	*Pseudomonas aeruginosa*	P3-S12	ST253	SAMN19112906	GWHBFUJ00000000
	B	Sputum	2019/3/27	Cerebral infarction	*Staphylococcus epidermidis*				
	B	Sputum	2019/3/27	Cerebral infarction	*Pseudomonas aeruginosa*	P3-S13	ST253	SAMN19112907	GWHBFUK00000000
	B	Sputum	2019/3/27	Cerebral infarction	*Pseudomonas aeruginosa*	P3-S14	ST253	SAMN19112908	GWHBFUL00000000
	B	Sputum	2019/4/23	Cerebral infarction	*Pseudomonas aeruginosa*	P3-S15	ST253	SAMN19112909	GWHBFUM00000000
	B	Blood	2019/5/7	Cerebral infarction	*Staphylococcus saprophyticus*				
	B	Blood	2019/5/27	Cerebral infarction	*Pseudomonas aeruginosa*	P3-S16	ST253	SAMN19112910	GWHBFUN00000000
P4	C	Sputum	2017/12/8	Severe head injury	*Pseudomonas aeruginosa*	P4-S1	ST277	SAMN19112911	GWHBFUO00000000

(Male,86)	C	Sputum	2018/1/5	Severe head injury	*Pseudomonas aeruginosa*	P4-S2	ST277	SAMN19112912	GWHBFUP00000000
	C	Sputum	2018/1/26	Severe head injury	*Pseudomonas aeruginosa*	P4-S3	ST277	SAMN19112913	GWHBFUQ00000000
	C	Sputum	2018/1/26	Severe head injury	*Pseudomonas aeruginosa*	P4-S4	ST277	SAMN19112914	GWHBFUR00000000
	C	Sputum	2018/1/26	Severe head injury	*Pseudomonas aeruginosa*	P4-S5	ST277	SAMN19112915	GWHBFUS00000000
	A	Sputum	2018/1/30	Lung infection	*Pseudomonas aeruginosa*	P4-S6	ST277	SAMN19112916	GWHBFUT00000000
	A	Sputum	2018/2/12	Lung infection	*Pseudomonas aeruginosa*	P4-S7	ST277	SAMN19112917	GWHBFUU00000000
	A	Sputum	2018/2/12	Lung infection	*Pseudomonas aeruginosa*	P4-S8	ST277	SAMN19112918	GWHBFUV00000000
	A	Sputum	2018/2/12	Coronary Heart Disease, Cerebral infarction	*Enterobacter cloacae*				
	A	Sputum	2018/2/22	Lung infection	*Pseudomonas aeruginosa*	P4-S9	ST277	SAMN19112919	GWHBFUW00000000
	A	Sputum	2018/2/26	Acute bronchitis	*Pseudomonas aeruginosa*	P4-S10	ST277	SAMN19112920	GWHBFUX00000000
	A	Sputum	2018/3/16	Acute bronchitis	*Escherichia coli*				
	B	Sputum	2017/8/22	Chronic bronchitis, Cerebral infarction	*Pseudomonas aeruginosa*	P5-S1	ST270	SAMN19112921	GWHBFUY00000000
	B	Sputum	2017/9/8	Chronic bronchitis	*Pseudomonas aeruginosa*	P5-S2	ST270	SAMN19112922	GWHBFUZ00000000
	B	Sputum	2017/9/15	Chronic bronchitis	*Pseudomonas aeruginosa*	P5-S3	ST270	SAMN19112923	GWHBFVA00000000
	B	Sputum	2017/10/20	Chronic bronchitis	*Pseudomonas aeruginosa*	P5-S4	ST270	SAMN19112924	GWHBFVB00000000
	B	Sputum	2017/10/20	Chronic bronchitis	*Pseudomonas aeruginosa*	P5-S5	ST270	SAMN19112925	GWHBFVC00000000
	B	Sputum	2017/10/30	Chronic bronchitis, Cerebral infarction	*Pseudomonas aeruginosa*	P5-S6	ST270	SAMN19112926	GWHBFVD00000000
	B	Sputum	2017/11/14	Chronic bronchitis, Cerebral infarction	*Pseudomonas aeruginosa*	P5-S7	ST270	SAMN19112927	GWHBFVE00000000
	B	Sputum	2017/11/23	Chronic bronchitis, Cerebral infarction	*Pseudomonas aeruginosa*	P5-S8	ST274	SAMN19112928	GWHBFVF00000000
	B	Sputum	2017/12/15	Chronic bronchitis, Cerebral infarction	*Proteus mirabilis*				
	B	Sputum	2017/12/26	Chronic bronchitis, Cerebral infarction	*Proteus mirabilis*				
	B	Sputum	2018/1/11	Chronic bronchitis, Cerebral infarction	*Proteus mirabilis*				
P5 (Female,80)	B	Sputum	2018/2/22	Cerebral infarction	*Pseudomonas aeruginosa*	P5-S9	ST274	SAMN19112929	GWHBFVG00000000
	B	Sputum	2018/3/16	Chronic bronchitis, Cerebral infarction	*Pseudomonas aeruginosa*	P5-S10	ST314	SAMN19112930	GWHBFVH00000000
	B	Sputum	2018/5/18	Chronic bronchitis	*Pseudomonas aeruginosa*	P5-S11	ST274	SAMN19112931	GWHBFVI00000000
	B	Sputum	2018/6/8	Chronic bronchitis	*Pseudomonas aeruginosa*	P5-S12	ST274	SAMN19112932	GWHBFVJ00000000
	B	Sputum	2018/7/13	Chronic bronchitis	*Pseudomonas aeruginosa*	P5-S13	ST274	SAMN19112933	GWHBFVK00000000
	B	Sputum	2018/8/2	Chronic bronchitis	*Klebsiella pneumoniae*				
	B	Sputum	2018/11/6	Chronic bronchitis	*Pseudomonas aeruginosa*	P5-S14	ST274	SAMN19112934	GWHBFVL00000000
	B	Sputum	2018/11/28	Chronic bronchitis	*Klebsiella pneumoniae*				
	B	Blood	2019/2/1	Chronic bronchitis	*Staphylococcus hominis*				
	B	Sputum	2019/2/1	Chronic bronchitis	*Pseudomonas aeruginosa*	P5-S15	ST314	SAMN19112935	GWHBFVM00000000
	B	Throat swab	2019/2/1	Chronic bronchitis	*Pseudomonas aeruginosa*	P5-S16	ST314	SAMN19112936	GWHBFVN00000000
	B	Throat swab	2019/2/22	Chronic bronchitis	*Pseudomonas aeruginosa*	P5-S17	ST274	SAMN19112937	GWHBFVO00000000
P6 (Female,83)	B	Urine	2017/10/20	type 2 diabetes mellitus	*Proteus vulgaris*				
	B	Urine	2017/11/14	type 2 diabetes mellitus, Coronary Heart Disease, Hyperlipidemia, Hypothyroidism	*Escherichia coli*				

	B	Urine	2017/11/23	type 2 diabetes mellitus, Coronary Heart Disease, Hyperlipidemia, Hypothyroidism	*Escherichia coli*				
	B	Urine	2017/12/8	type 2 diabetes mellitus, Coronary Heart Disease, Hyperlipidemia, Hypothyroidism	*Pseudomonas aeruginosa*	P6-S1	ST231	SAMN19 112938	GWHBFVP 00000000
	B	Urine	2017/12/21	type 2 diabetes mellitus, Coronary Heart Disease, Hyperlipidemia, Hypothyroidism	*Proteus vulgaris*				
	B	Urine	2017/12/26	type 2 diabetes mellitus, Coronary Heart Disease, Hyperlipidemia, Hypothyroidism	*Proteus vulgaris*				
	B	Urine	2018/1/11	type 2 diabetes mellitus, Coronary Heart Disease	*Proteus vulgaris*				
	B	Urine	2018/6/8	type 2 diabetes mellitus	*Escherichia coli*				
	B	Sputum	2018/8/2	type 2 diabetes mellitus	*Pseudomonas aeruginosa*	P6-S2	ST231	SAMN19 112939	GWHBFVQ 00000000
	B	Sputum	2018/8/2	type 2 diabetes mellitus	*Pseudomonas aeruginosa*	P6-S3	ST231	SAMN19 112940	GWHBFVR 00000000
	B	Sputum	2018/8/7	type 2 diabetes mellitus	*Pseudomonas aeruginosa*	P6-S4	ST231	SAMN19 112941	GWHBFVS 00000000
	B	Sputum	2018/8/7	type 2 diabetes mellitus	*Pseudomonas aeruginosa*	P6-S5	ST231	SAMN19 112942	GWHBFVT 00000000
	B	Sputum	2018/8/14	type 2 diabetes mellitus	*Pseudomonas aeruginosa*	P6-S6	ST231	SAMN19 112943	GWHBFVU 00000000
	B	Blood	2018/8/14	type 2 diabetes mellitus	*Staphylococcus hominis*				
	B	Sputum	2018/8/27	type 2 diabetes mellitus	*Enterococcus faecalis*				
P7 (Male,94)	A	Sputum	2017/8/22	Acute exacerbation of chronic bronchitis	*Pseudomonas aeruginosa*	P7-S1	ST640	SAMN19 112944	GWHBFVV 00000000
	A	Sputum	2017/8/31	Acute exacerbation of chronic bronchitis	*Pseudomonas aeruginosa*	P7-S2	ST640	SAMN19 112945	GWHBFV W00000000
	A	Sputum	2017/9/15	Acute exacerbation of chronic bronchitis	*Acinetobacter baumannii*				
	A	Sputum	2017/9/22	Acute exacerbation of chronic bronchitis	*Acinetobacter baumannii*				
	A	Sputum	2017/10/13	Pneumonia	*Escherichia coli*				
	A	Sputum	2017/10/13	Acute exacerbation of chronic bronchitis	*Pseudomonas aeruginosa*	P7-S3	ST640	SAMN19 112946	GWHBFVX 00000000
	A	Sputum	2017/10/30	Pneumonia	*Escherichia coli*				
	A	Sputum	2017/10/30	Pneumonia	*Escherichia coli*				
	A	Sputum	2017/11/14	Pneumonia	*Escherichia coli*				
	A	Sputum	2017/11/23	Pneumonia	*Staphylococcus aureus*				
	A	Sputum	2017/12/1	Acute exacerbation of chronic bronchitis	*Staphylococcus aureus*				
	A	Sputum	2017/12/8	Acute exacerbation of chronic bronchitis	*Pseudomonas aeruginosa*	P7-S4	ST640	SAMN19 112947	GWHBFVY 00000000
	A	Sputum	2017/12/15	Acute exacerbation of chronic bronchitis	*Staphylococcus aureus*				
	A	Sputum	2017/12/26	Acute exacerbation of chronic bronchitis	*Klebsiella pneumoniae*				
	A	Sputum	2018/1/5	Acute exacerbation of chronic bronchitis	*Klebsiella pneumoniae*				
	A	Sputum	2018/1/26	Acute exacerbation of chronic bronchitis	*Acinetobacter lwoffii*				
	A	Throat swab	2018/1/30	Acute exacerbation of chronic bronchitis	*Streptococcus agalactiae，GBS*				
	A	Throat swab	2018/3/16	Acute exacerbation of chronic bronchitis	*Streptococcus agalactiae，GBS*				

	A	Sputum	2018/4/8	Acute exacerbation of chronic bronchitis	*Pseudomonas aeruginosa*	P7-S5	ST640	SAMN19 112948	GWHBFVZ 00000000
	A	Sputum	2018/4/8	Acute exacerbation of chronic bronchitis	*Pseudomonas aeruginosa*	P7-S6	ST640	SAMN19 112949	GWHBFWA 00000000
	A	Sputum	2018/4/8	Acute exacerbation of chronic bronchitis	*Klebsiella pneumoniae*				

Supplementary material Table S2. Antimicrobial susceptibility testing of the *P. aeruginosa* isolates from the inpatients. The minimal inhibitory concentrations (MICs) of aztreonam (AZT), cefepime (FEP), cefoperazone (CFP), ceftazidime (CAZ), ceftriaxone (CTR); imipenem (IMI), piperacillin (PRL), ticarcillin (TIC), ticarcillin/clavulanate (T/C), chloramphenicol (CHL), gentamicin (GEN), levofloxacin (LEV), tobramycin (TOB), amikacin (AMI) were shown. MLST, multilocus sequence typing. ID, Identifier.

Patient ID	Sample ID	MLST type	MIC (μg/mL)													
			**AZT**	**FEP**	**CFP**	**CAZ**	**CTR**	**IMI**	**PRL**	**TIC**	**T/C**	**CHL**	**GEN**	**LEV**	**TOB**	**AMI**
P1	P1-S1	385	2	≤8	≤16	≤1	32	≤4	≤16	≤16	≤16	>16	≤4	≤2	≤4	≤16
	P1-S2	385	16	≤8	≤16	8	32	≤4	≤16	64	64	>16	≤4	≤2	≤4	≤16
	P1-S3	385	16	≤8	≤16	8	32	>8	≤16	64	64	>16	≤4	>4	≤4	≤16
	P1-S4	385	16	≤8	>32	8	32	≤4	>64	>64	64	>16	8	>4	≤4	≤16
	P1-S5	385	16	≤8	32	8	32	8	64	>64	>64	>16	≤4	>4	≤4	≤16
	P1-S6	385	16	16	>32	>16	>32	8	>64	>64	>64	>16	≤4	≤2	≤4	≤16
	P1-S7	385	>16	>16	>32	>16	>32	8	>64	>64	>64	>16	≤4	≤2	≤4	≤16
	P1-S8	385	16	≤8	≤16	8	32	>8	≤16	64	64	>16	≤4	>4	≤4	≤16
	P1-S9	385	>16	≤8	32	8	32	≤4	≤16	>64	>64	>16	≤4	>4	≤4	≤16
	P1-S10	385	>16	16	>32	>16	>32	≤4	>64	>64	>64	>16	≤4	≤2	≤4	≤16
	P1-S11	385	>16	16	≤16	8	32	8	≤16	64	64	>16	>8	>4	>8	>32
	P1-S12	385	16	≤8	32	8	32	8	≤16	>64	>64	>16	≤4	>4	≤4	≤16
	P1-S13	385	8	≤8	≤16	2	8	≤4	≤16	64	64	>16	≤4	>4	≤4	≤16
	P1-S14	385	8	≤8	≤16	2	32	8	≤16	64	64	>16	≤4	>4	≤4	≤16
	P1-S15	385	8	≤8	≤16	2	8	>8	≤16	64	64	>16	≤4	>4	≤4	≤16
P2	P2-S1	314	8	≤8	≤16	2	>32	≤4	≤16	>64	64	>16	≤4	≤2	≤4	≤16
P3	P3-S1	110	16	≤8	≤16	8	>32	≤4	≤16	64	>64	>16	≤4	≤2	≤4	≤16
	P3-S2	110	16	≤8	≤16	8	32	≤4	≤16	64	64	>16	≤4	≤2	≤4	≤16
	P3-S3	110	>16	16	>32	16	32	≤4	>64	>64	>64	>16	≤4	>4	≤4	≤16
	P3-S4	110	16	≤8	32	8	>32	8	64	>64	>64	>16	≤4	≤2	≤4	≤16
	P3-S5	110	16	≤8	≤16	<8	32	8	64	64	>64	>16	8	≤2	≤4	≤16
	P3-S6	253	8	≤8	≤16	2	32	≤4	≤16	64	>64	>16	≤4	≤2	≤4	≤16
	P3-S7	2326	2	≤8	≤16	≤1	>32	≤4	≤16	≤16	≤16	16	≤4	≤2	≤4	≤16
	P3-S8	253	8	≤8	≤16	2	32	≤4	≤16	64	64	>16	≤4	≤2	≤4	≤16
	P3-S9	253	8	≤8	≤16	≤1	32	≤4	≤16	64	64	>16	≤4	≤2	≤4	≤16
	P3-S10	253	>16	≤8	>32	8	>32	≤4	>64	>64	>64	≥16	≤4	>4	≤4	≤16
	P3-S11	253	16	≤8	>32	16	>32	≤4	64	64	>64	≥16	≤4	≤2	≤4	≤16
	P3-S12	253	16	≤8	>32	16	>32	≤4	64	64	>64	≥16	≤4	≤2	≤4	≤16
	P3-S13	253	≤1	≤8	≤16	≤1	≤1	≤4	64	>64	64	16	>8	≤2	>8	≤16
	P3-S14	253	8	≤8	32	8	>32	8	64	64	64	>16	≤4	≤2	≤4	≤16
	P3-S15	253	16	≤8	32	16	>32	8	64	64	>64	≥16	≤4	≤2	≤4	≤16
P4	P4-S1	277	>16	≤8	32	8	>32	≤4	64	>64	>64	>16	≤4	>4	≤4	≤16
	P4-S2	277	16	≤8	≤16	8	>32	≤4	≤16	>64	>64	>16	≤4	>4	≤4	≤16
	P4-S3	277	16	≤8	≤16	8	>32	≤4	≤16	>64	>64	>16	≤4	>4	≤4	≤16

	P4-S4	277	16	≤8	≤16	8	>32	≤4	≤16	>64	>64	>16	≤4	>4	≤4	≤16
	P4-S5	277	>16	16	>32	>16	≥32	≤4	>64	>64	>64	>16	≤4	>4	≤4	≤16
	P4-S6	277	>16	≤8	>32	16	>32	>8	>64	>64	>64	>16	≤4	>4	≤4	≤16
	P4-S7	277	>16	≤8	>32		>32	≤4	64	>64	>64	>16	≤4	>4	≤4	≤16
	P4-S8	277	>16	≤8	>32	>16	>32	≤4	64	>64	>64	>16	≤4	4	≤4	≤16
	P4-S9	277	16	≤8	32	8	≥32	≤4	64	64	>64	>16	≤4	4	≤4	≤16
	P4-S10	277	16	≤8	≤16	8	32	≤4	≤16	>64	>64	>16	≤4	>4	≤4	≤16
P5	P5-S1	270	8	≤8	≤16	2	>32	≤4	≤16	64	64	>16	≤4	≤2	≤4	≤16
	P5-S2	270	8	≤8	≤16	2	32	≤4	64	≤16	≤16	>16	≤4	≤2	≤4	≤16
	P5-S3	270	8	≤8	≤16	2	32	≤4	≤16	≤16	≤16	>16	≤4	≤2	≤4	≤16
	P5-S4	270	8	≤8	≤16	2	>32	≤4	≤16	≤16	64	>16	≤4	≤2	≤4	≤16
	P5-S5	270	8	≤8	≤16	8	>32	≤4	64	64	64	>16	≤4	≤2	≤4	≤16
	P5-S6	270	8	≤8	≤16	8	>32	≤4	≤16	64	64	>16	≤4	≤2	≤4	≤16
	P5-S7	270	8	≤8	≤16	2	>32	≤4	≤16	≤16	64	>16	≤4	≤2	≤4	≤16
	P5-S8	274	2	≤8	≤16	2	>32	>8	64	64	64	>16	≤4	≤2	≤4	≤16
	P5-S9	274	8	≤8	≤16	8	>32	≤4	≤16	64	64	>16	≤4	≤2	≤4	≤16
	P5-S10	314	8	≤8	≤16	2	>32	≤4	≤16	≤16	64	>16	8	≤2	≤4	≤16
	P5-S11	274	8	≤8	≤16	8	>32	≤4	≤16	64	64	>16	≤4	≤2	≤4	≤16
	P5-S12	274	8	≤8	>32	8	>32	≤4	>64	64	64	>16	≤4	≤2	≤4	≤16
	P5-S13	274	8	≤8	≤16	8	>32	>8	64	64	64	>16	≤4	≤2	≤4	≤16
	P5-S14	274	8	≤8	≤16	8	>32	≤4	64	64	64	>16	≤4	≤2	≤4	≤16
	P5-S15	314	>16	16	>32	>16	>32	>8	>64	>64	>64	>16	>8	≤2	>8	32
	P5-S16	314	8	≤8	≤16	8	>32	≤4	≤16	64	64	>16	≤4	≤2	≤4	≤16
	P5-S17	274	16	≤8	>32	>16	>32	8	64	>64	>64	>16	≤4	≤2	≤4	≤16
P6	P6-S1	231	8	≤8	≤16	2	32	≤4	≤16	64	64	>16	≤4	≤2	≤4	≤16
	P6-S2	231	16	≤8	≤16	8	32	>8	≤16	64	64	>16	≤4	≤2	≤4	≤16
	P6-S3	231	8	≤8	≤16	2	32	≤4	≤16	64	64	>16	≤4	≤2	≤4	≤16
	P6-S4	231	8	≤8	≤16	2	32	≤4	≤16	64	64	>16	≤4	≤2	≤4	≤16
	P6-S5	231	8	≤8	≤16	8	32	≤4	≤16	64	64	>16	≤4	4	≤4	≤16
	P6-S6	231	>16	≤8	32	8	>32	≤4	≤16	>64	>64	>16	≤4	>4	≤4	≤16
P7	P7-S1	640	16	≤8	≤16	8	32	8	≤16	>64	>64	>16	≤4	>4	≤4	≤16
	P7-S2	640	16	≤8	≤16	8	32	8	≤16	>64	>64	>16	≤4	>4	≤4	≤16
	P7-S3	640	16	≤8	32	8	>32	>8	≤16	>64	>64	>16	≤4	>4	≤4	≤16
	P7-S4	640	≤1	≤8	≤16	≤1	8	>8	≤16	≤16	≤16	>16	≤4	≤2	≤4	≤16
	P7-S5	640	8	≤8	≤16	2	32	>8	≤16	64	64	>16	≤4	≤2	≤4	≤16

Supplementary material Table S3. Recurrently mutated genes and mutations. Recurrently mutated genes were defined as genes contained intra-clonal variations in ≥2 *P. aeruginosa* isolates. The annotations of the mutations was obtained by using SnpEff with the PAO1 strain (GenBank accession NC_002516) as the reference genome; function classification and pathway of the genes were obtained from the Pseudomonas genome database, PseudoCAP (https://www.pseudomonas.com/pseudocap).

Gene	Mutations		Isolate identifier (MLST clonal type)	Product description[a]	Functional Classification[b]	Pathway[c]
fptA	788A>G	p.Gln263Arg	P1-S4 (ST385)	Fe(III)-pyochelin outer membrane receptor precursor	Transport of small molecules	-
	†2093C>T	p.Ser698Phe	P1-S13, P1-S15 (ST385); P3-S2 (ST110) ; P5-S1, P5-S2, P5-S5, P5-S6, P5-S7 (ST270); P3-S9, P3-S11 (ST253)			
pvdS	224C>T	p.Ala75Val	P1-S13 (ST385)	extracytoplasmic-function sigma-70 factor	Transcriptional regulators	-
	239G>A	p.Arg80His	P1-S14 (ST385)			
	97A>G	p.Thr33Ala	P3-S4 (ST110)			
	139T>C	p.Phe47Leu	P3-S1, P3-S2, P3-S3, P3-S5 (ST110)			
	104G>A	p.Cys35Tyr	P3-S10 (ST253)			
	140T>G	p.Phe47Cys	P3-S12, P3-S13, P3-S14, P3-S15, P3-S16 (ST253)			
	223G>A	p.Ala75Thr	P3-S8 (ST253)			
	115G>A	p.Ala39Thr	P5-S9, P5-S11, P5-S12, P5-S13, P5-S14 (ST274)			
mexZ	1G>C	p.Val1Leu	P1-S6, P1-S7[c], P1-S10 (ST385)	multidrug efflux protein	Transcriptional regulators	beta-Lactam resistance
	452C>T	p.Pro151Leu	P3-S1, P3-S2, P3-S3, P3-S5 (ST110)			
	515G>A	p.Gly172Asp	P4-S10 (ST277)			
lasR	571G>A	p.Gly191Ser	P3-S4 (ST110)	transcriptional regulator LasR	Adaptation, Protection	Quorum sensing；Biofilm formation - Pseudomonas aeruginosa
	692C>T	p.Ala231Val	P5-S3, P5-S4, P5-S5, P5-S6, P5-S7 (ST270)			
	555G>T	p.Leu185Phe	P5-S9, P5-S11, P5-S12, P5-S13, P5-S14 (ST274)			
	670C>T	p.Arg224Cys	P5-S17 (ST274)			
aguA	118G>A	p.Gly40Ser	P3-S1 (ST110)	agmatine deiminase	Amino acid biosynthesis and metabolism	Metabolic pathways;L-arginine degradation IV (arginine decarboxylase/agmatine deiminase pathway);arginine degradation II; Arginine and proline metabolism
	-1009G>A		P4-S10 (ST277)			
ampD	58A>G	p.Asn20Asp	P5-S15 (ST314)	N-acetyl-anhydromuranmyl-L-alanine amidase	Cell wall / LPS / capsule	-
	229C>T	p.His77Tyr	P3-S11 (ST253)			
	466G>A	p.Gly156Ser	P3-S12,P3-S13,P3-S14,P3-S15,P3-S16 (ST253)			
fha1	393G>A	p.Arg131Arg	P4-S7,P4-S8,P4-S10 (ST277)	Fha domain-containing protein	Protein secretion/export apparatus	Biofilm formation - Pseudomonas aeruginosa; Bacterial secretion system
	1031T>C	p.Val344Ala	P3-S3 (ST110)			
fleQ	302T>G	p.Val101Gly	P1-S1 (ST385)	transcriptional	Transcriptional	Two-component

	347A>G	p.Asp116Gly	P4-S7,P4-S8,P4-S10 (ST277)	regulator FleQ	regulators; Motility & Attachment	system;Biofilm formation - Pseudomonas aeruginosa
fruK	214A>G	p.Met72Val	P4-S3 (ST277)	1-phosphofructokinase	Transport of small molecules; Central intermediary metabolism	Galactose metabolism; Fructose and mannose metabolism
	941C>T	p.Ala314Val	P3-S1 (ST385)			
mdlC	630C>T	p.Asp210Asp	P3-S1 (ST110)	benzoylformate decarboxylase	Carbon compound catabolism	Microbial metabolism in diverse environments;Aromatic compound catabolism;Aminobenzoate degradation;4-hydroxymandelate degradation
	908G>A	p.Gly303Asp	P1-S10 (ST385)			
oprD	†831G>A	p.Trp277*	P3-S4 (ST110)	porin D	Transport of small molecules	Two-component system; beta-Lactam resistance
	†1017G>A	p.Trp339*	P1-S11,P1-S13,P1-S14,P1-S15 (ST385)			
pelB	55T>C	p.Trp19Arg	P4-S8 (ST277)	pellicle/biofilm biosynthesis protein PelB	Cell wall / LPS / capsule	Biofilm formation - Pseudomonas aeruginosa
	2345C>T	p.Thr782Ile	P3-S1 (ST110)			
pvdL	3357C>T	p.Gly1119Gly	P4-S10 (ST277)	peptide synthase	Adaptation, Protection	Pyoverdine synthesis
	4681G>C	p.Val1561Leu	P3-S12,P3-S13,P3-S14,P3-S15,P3-S16 (ST253)			
wbpM	521A>G	p.Asn174Ser	P4-S10 (ST277)	nucleotide sugar epimerase/dehydratase WbpM	Cell wall / LPS / capsule; Putative enzymes; Membrane proteins	UDP-N-acetyl-alpha-D-fucosamine biosynthesis; Lipopolysaccharide biosynthesis
	634A>G	p.Lys212Glu	P6-S1 (ST231)			
laoA	855C>T	p.Ser285Ser	P4-S10 (ST277)	LaoA	Putative enzymes	-
	1037T>C	p.Leu346Pro	P3-S5 (ST110)			
PA1503	53C>T	p.Ala18Val	P3-S2 (ST110)	hypothetical protein	Hypothetical, unclassified, unknown	-
	128A>G	p.Gln43Arg	P3-S3 (ST110)			
	137A>G	p.Asp46Gly	P4-S7 (ST277)			
PA2314	280C>T	p.Leu94Leu	P4-S6 (ST277)	major facilitator superfamily transporter	Transport of small molecules; Membrane proteins	-
	637C>A	p.Arg213Ser	P5-S9,P5-S11,P5-S12,P5-S13,P5-S14 (ST274)			
mexS	88G>T	p.Glu30*	P6-S2,P6-S3,P6-S4 (ST231)	MexS, MexS is a suppressor of MexT.	Transcriptional regulators; Putative enzymes	-
	697G>T	p.Val233Phe	P5-S8,P5-S17 (ST274)			
tse5	677A>G	p.Tyr226Cys	P3-S4 (ST110)	tse5	Hypothetical, unclassified, unknown	-
	2714C>T	p.Ala905Val	P4-S3,P4-S6,P4-S7,P4-S8,P4-S9,P4-S10 (ST277)			
PA3215	-2088G>A		P5-S5,P5-S6,P5-S7 (ST270)	transcriptional regulator	Transcriptional regulators	-
	-2152C>T		P4-S3 (ST277)			
PA3491	685G>A	p.Val229Ile	P4-S7,P4-S9,P4-S10 (ST277)	electron transport complex subunit C	Membrane proteins; Energy metabolism	-
	1447G>A	p.Ala483Thr	P3-S5 (ST110)			
PA3623	131G>A	p.Gly44Asp	P1-S14 (ST385)	hypothetical protein	Hypothetical, unclassified, unknown	-
	645T>C	p.Gly215Gly	P4-S10 (ST277)			
PA4039	529G>A	p.Val177Ile	P1-S11,P1-S13,P1-S14,P1-S15 (ST385)	hypothetical protein	Hypothetical, unclassified, unknown	-
	1230G>C	p.Gly410Gly	P3-S2 (ST110)			
PA4523	1396C>T	p.Leu466Phe	P3-S3 (ST110)	hypothetical protein	Hypothetical, unclassified, unknown	-
	1618G>T	p.Glu540*	P5-S16 (ST314)			

[a]Product description reported on NCBI and PseudoCAP.
[b]Kyoto Encyclopedia of Genes and Genomes (KEGG) pathway assigned in PseudoCAP.
[c]The frequency of reads for the mutated alleles was 0.65, while others were ≥0.9.
[†]Mutations that are identified in the public *P. aeruginosa* gene sequences. 2093C>T in *fptA*, strain DVT419 (GenBank accession NZ_CP050328.1) and strain T2101 (NZ_CP039990.1). 831G>A in *oprD*, strain IRP41 (GenBank accession MT293231.1). 1017G>A in *oprD*, strain FDAARGOS_570 (GenBank accession NZ_CP033835.1)

Supplementary material Table S4. Summary of structural variations. Structural variations (SVs) were identified by using Pindel and DELLY, with the PAO1 strains as reference genomes. The SVs were also manually examined by aligning *de novo* assemblies of isolates to reference genome with BLAST (blastn). SVs were validated if assemblies had contigs covering the junction sites of the SVs.

P. aeruginosa isolate ID	Type of SV	Start position (bp)	End position (bp)	length (bp)	No. of genes in the SV	Genes	Reads no. supporting the allele[a]	Is SV in assembly
P1-S1	deletion	1556970	1575650	18680	18	partial of PA1429, lasR, rsaL, lasI, PA1433, PA1434, PA1435, PA1436, PA1437, PA1438, PA1439, PA1440, PA1441, PA1442, fliM, fliN, fliO, fliP, fliQ, partial of fliR	148	yes
P1-S13	deletion	4159790	4166586	6796	7	partial of spdH,PA3714, PA3715, PA3716, PA3717, PA3718, armR, PA3720, partial of nalC	204	yes
P1-S14	deletion	4159790	4166586	6796	7	partial of spdH, PA3714, PA3715, PA3716, PA3717, PA3718, armR, PA3720, partial of nalC	131	yes
P1-S14	deletion	2459718	2459949	231	0	partial of PA2235	122	yes
P4-S5	deletion	5039562	5072768	33206	26	partial of opdP, PA4502, PA4503, PA4504, PA4505, PA4506, PA4507, PA4508, PA4509, PA4510, PA4511, lpxO1, PA4513, PA4514, PA4515, PA4516, PA4517, PA4518, speC, PA4520, PA4521, ampD, PA4523, nadC, PA4524.1, pilA, pilB, partial of pilD	128	yes
P5-S16	deletion	3467260	3480879	13619	15	partial of PA3089, PA3089a, PA3091, fadH1, PA3093, PA3094, PA3094.1, PA3094.2, PA3094.3, xcpZ, xcpY, xcpX, xcpW, xcpV, xcpU, xcpT, partial of xcpS	138	yes
P6-S2	deletion	1575528	1575581	53	0	partial of fliQ, partial of fliR	120	yes
P6-S3	deletion	1575528	1575581	53	0	partial of fliQ, partial of fliR	148	yes
P6-S4	deletion	1575528	1575581	53	0	partial of fliQ, partial of fliR	124	yes
P6-S5	deletion	1575528	1575581	53	0	partial of fliQ, partial of fliR	145	yes
P6-S6	deletion	1575528	1575581	53	0	partial of fliQ, partial of fliR	112	yes
P7-S4	deletion	2412143	2413114	971	1	hcnA, partial of hcnB	56	yes

[a]The number of reads supporting the SV allele, reported by Pindel.
